# What Can Current Biomarker Data Tell Us About the Risks of Lung Cancer Posed by Heated Tobacco Products?

**DOI:** 10.1093/ntr/ntad081

**Published:** 2023-05-21

**Authors:** Sophie Braznell, John Campbell, Anna B Gilmore

**Affiliations:** Tobacco Control Research Group, Department for Health, University of Bath, Bath, UK; Department for Health, University of Bath, Bath, UK; Tobacco Control Research Group, Department for Health, University of Bath, Bath, UK; SPECTRUM (Shaping Public Health Policies to Reduce Inequalities and Harm) Consortium, Bath, UK

## Abstract

**Introduction:**

Heated tobacco products (HTPs) are marketed as less harmful alternatives to cigarettes, but the lung cancer risk of HTPs is unknown. In the absence of epidemiological data, assessing the risks of HTPs relies on biomarker data from clinical trials. This study examined existing biomarker data to determine what it tells us about the lung cancer risk posed by HTPs.

**Aims and Methods:**

We identified all biomarkers of exposure and potential harm measured in HTP trials and evaluated their appropriateness based on ideal characteristics for measuring lung cancer risk and tobacco use. The effects of HTPs on the most appropriate biomarkers within cigarette smokers switched to HTPs and compared to continued cigarette smoking or cessation were synthesized.

**Results:**

Sixteen out of eighty-two biomarkers (7 exposure and 9 potential harm) measured in HTP trials have been associated with tobacco use and lung cancer, dose-dependently correlated with smoking, modifiable upon cessation, measured within an appropriate timeframe, and had results published. Three of the exposure biomarkers significantly improved in smokers who switched to HTPs and were not significantly different from cessation. The remaining 13 biomarkers did not improve—in some instances worsening upon switching to HTPs—or were inconsistently affected across studies. There were no appropriate data to estimate the lung cancer risk of HTPs in non-smokers.

**Conclusions:**

The appropriateness of existing biomarker data in assessing lung cancer risk of HTPs, both relative to cigarettes and their absolute risk, is limited. Furthermore, findings on the most appropriate biomarkers were conflicting across studies and largely showed no improvement following a switch to HTPs.

**Implications:**

Biomarker data are fundamental to assessing the reduced risk potential of HTPs. Our evaluation suggests much of the existing biomarker data on HTPs is inappropriate for determining the risk of lung cancer posed by HTPs. In particular, there is a paucity of data on the absolute lung cancer risk of HTPs, which could be obtained from comparisons to smokers who quit and never smokers exposed to or using HTPs. There is an urgent need for further exploration of the lung cancer risks posed by HTPs, via clinical trials and, in the long-term, confirmation of these risks via epidemiological studies. However, careful consideration should be given to biomarker selection and study design to ensure both are appropriate and will provide valuable data.

## Introduction

Lung cancer is the most common type of cancer and leading cause of cancer death worldwide. It is estimated that there were 2.09 million new cases (11.6% of total cancer cases) and 1.76 million deaths (18.4% of total cancer deaths) in 2018.^1^ It is particularly prevalent among tobacco users and is the third-leading cause of deaths in this group.^2^ In the United Kingdom, over 70% of lung cancer cases are attributed to either active smoking (71%) or secondhand exposure (1%).^3^ Smoking cessation decreases the risk of lung cancer.^4^ Altogether, this indicates the majority of lung cancer is preventable, specifically by eliminating tobacco exposure.^5^

Heated tobacco products (HTPs) heat tobacco to produce nicotine-containing inhalable emissions. In heating tobacco at lower temperatures than combustible cigarettes HTPs are designed to reduce the amounts of harmful chemicals emitted compared to combustible cigarettes, and therefore, in theory, reduce the risks of harm and diseases, like lung cancer. Manufacturers, namely transnational tobacco companies, market HTPs as less harmful alternatives to cigarettes,^6^ which recent evidence suggests contributes to consumer uptake.^7–9^ Indeed, HTP use is increasing worldwide.^10–12^

It is difficult to ascertain the relationship between HTPs and lung cancer due to: (1) the lack of post-market epidemiological studies measuring disease incidence; (2) the long latency period between tobacco exposure and lung cancer development; and (3) difficulties in attributing disease manifestation to HTPs in former or current smokers. Therefore, in the absence of epidemiological data, data from clinical trials, particularly on biomarkers, form the majority of clinical evidence to date on the health risks of HTPs.^13,14^ The findings from these trials are increasingly significant as they are used by the industry to substantiate its claim that HTPs are less harmful than cigarettes,^15–17^ and by public health policy makers to make important tobacco control policy decisions.^15,18–20^ Given the prevalence of lung cancer among smokers, investigating the risk of lung cancer is an important element in assessing the potential health risks or benefits of HTPs.

In a recent systematic review of clinical trials on HTPs, we found a wide variety of biomarkers had been measured in individuals exposed to HTPs,^21^ including biomarkers of exposure and potential harm (also known as biomarkers of effect).^22^ During the latency period between exposure and disease manifestation, such biomarkers are important in assessing the potential risks of novel tobacco products. However, it is imperative biomarkers with a strong biological rationale are chosen to ensure an accurate assessment.^23^

Others have previously examined proposed biomarkers for assessing the reduced risk potential of novel tobacco and nicotine products.^22,24–29^ However, these either pre-date the HTP clinical trials or were conducted by the tobacco industry, which has a long documented history of research manipulation.^30^ Additionally, the appropriateness of the biomarkers in assessing the lung cancer risk of HTPs has hitherto remained unexplored. Therefore, we aimed to examine the existing biomarker data on HTPs to (1) evaluate its appropriateness in estimating the risk of lung cancer posed by HTPs, and (2) determine what the most appropriate data tells us about the potential lung cancer risk of HTPs.

## Methods

We previously systematically reviewed 40 interventional clinical trials on HTPs published between January 2010 and April 2022. The method of literature selection and data collection has been described previously in the protocol^31^ and publication.^21^ In brief, trial characteristics and outcome data were extracted from interventional clinical trials of any design that assigned at least one group of adults a currently marketed HTP.

Thirty-five of these 40 trials measured biomarkers of exposure or potential harm. For this study, we obtained the following data on these trials, extracted under the previous review: study characteristics (design, participant arms, duration, mode of exposure), biomarkers measured and reported on in trial publications, biomarker type (exposure or potential harm), matrix the biomarker was measured in, and physiological or pathological process the biomarker measured (e.g. inflammation, oxidative stress, exposure, oral health, etc.). We did not obtain data on biomarkers specific to cardiovascular function with no known relation to lung cancer.

We first sought to evaluate the appropriateness of the biomarkers used in the 35 clinical trials in assessing the lung cancer risk of HTPs. Ideal characteristics for biomarkers of lung cancer risk and tobacco use have previously been proposed.^22,24,32,33^ These are summarized in Table 1.

**Table 1.** Ideal characteristics for biomarkers of lung cancer risk and tobacco use.

Chang et al.^22^	Atwater and Massion^32^	Shields^33^	Institute of Medicine^24^	Description
Plausibility/Coherence	Biologically plausible	–	Relevant to outcome of interest	Marker effects are reflective of the pathological process caused by exposure and do not seriously contradict existing knowledge on disease pathology.
Sensitivity	Accurate	Sensitivity	Sensitivity	How well the marker detects clinically relevant differences following exposure, that is, detect true positives.
Predictability	–	Type of measure (e.g. exposure, harm, dose)	Type of measure (e.g. exposure, harm, dose)	How well the marker predicts disease risk or is an adequate intermediate for risk.
Temporality	–	–	–	Exposure precedes effect on marker.
Experiment	–	Harm reduction in dose–response data	Harm reduction in dose–response data	Reducing exposure reverses, lessens or removes the observed effect on the marker.
Biological gradient	–	Dose–response data	Dose‐response data	Magnitude of exposure proportional to the magnitude of effect on marker.
Specificity	Accurate	Specificity	Specificity	Marker is specific to the exposure, and is not influenced by confounding variables, that is, detect true negatives.
Analogy	–	–	–	Relationship between marker effect and reduced disease risk has been demonstrated in similar exposures (e.g. environmental pollution).
Consistency	–	Reproducibility	Reproducibility	Relationship between exposure & effect repeatedly observed in different settings, for example, methods, investigators, participants, instruments.
–	–	Tissue assayed	Target tissue and outcome effect	Whether the marker is measured in target tissues (e.g. lung tissue) or surrogate tissues (e.g. blood).

Based on these proposed characteristics, we formulated the following questions to be addressed in our evaluation:

1. Has there been a significant association observed between the biomarker and:
  a. tobacco use?
  b. lung cancer?
2. Has a dose–response relationship been found between the biomarker and tobacco use?
3. Upon cigarette smoking cessation, does the effect on the biomarker deviate from the effect caused by continued cigarette smoking?
4. Have the HTP clinical trials that measured the biomarker reported results on the biomarker?
5. Has the biomarker been measured in at least one HTP clinical trial with a sufficient follow-up duration based on evidenced time to deviation following cigarette smoking cessation?
6. Has the biomarker been measured in non-smokers who were directly exposed to or used HTPs?

Specificity, reproducibility, temporality, and analogy were not explicitly examined. Specificity of biomarkers of exposure and potential harm has been discussed previously.^22,24,25^ Assessing reproducibility would require a comprehensive review of all studies to date to accurately determine whether biomarker effects are consistently observed, which was not feasible to conduct on each of the 82 biomarkers to be evaluated. Temporality is evident between smoking and biomarker effects—a biomarker effect is unlikely to precede initiation of cigarette consumption—and can be inferred from evidence of a dose-dependent correlation. Analogy was not assessed due to the existing breadth of our examination. It may not be necessary for a biomarker to literally reverse in smokers who switch to HTPs, but simply not continue on the same trajectory as continued cigarette smoking. Biomarkers that remain unchanged in smokers who quit but are significantly different from continued smokers may, therefore, still be useful in assessing the risks of HTPs. Hence, we examined deviation, not reversibility.

In order to address questions 1–3, we conducted rapid reviews of existing literature. To identify literature, we searched Google Scholar using strings comprising variations of the biomarker name (e.g. “Hemoglobin A1C” or “HbA1c” or “Glycated hemoglobin”) and terms specific to each of the questions. For 1a and 2, these included smoking-related terminology (e.g. “tobacco,” “smoking” or “cigarettes”). For 1b these included lung cancer-related terminology (e.g. “lung cancer,” “lung cancer risk” or “lung carcinoma”). For question 3, these included cessation terminology (e.g. “cessation,” “quit” or “abstinence”). We also searched reference lists of included literature. We did not enforce any eligibility criteria, instead opting to read as much literature as possible or required to answer each question. Clinical studies and systematic reviews of clinical data were prioritized. The findings from the rapid reviews were synthesized narratively.

Questions 4–6 were addressed using the data obtained from the previous systematic review of HTP clinical trials described above. Answers to each of the six questions were collated and presented in a heatmap.

Finally, we narratively synthesized the findings from the HTP clinical trials on the biomarkers identified as appropriate in the above evaluation. Specifically, we extracted and synthesized the effect of HTPs on the most appropriate biomarkers: between baseline and last follow-up in smokers assigned HTPs; between baseline and last follow-up in non-smokers assigned HTPs; between HTP and cigarette groups at last follow-up; and between HTP and cessation groups at last follow-up. Findings were only included from trials that were of sufficient duration in line with evidenced time to deviation. The findings and characteristics of studies included in this synthesis were tabulated.

## Results

### Evaluation of Biomarkers

**Figure 1.**
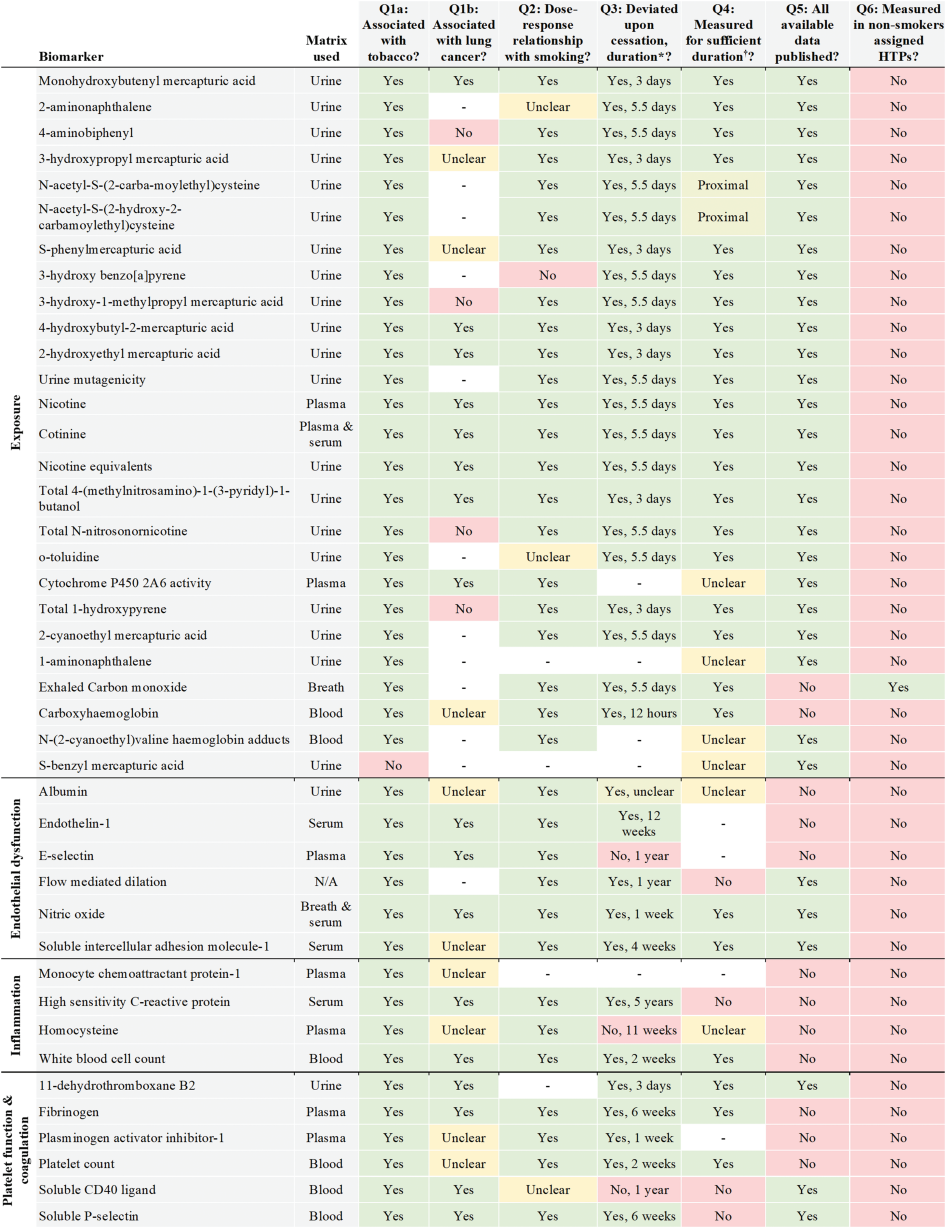
Summary of evaluation of biomarkers relating to exposure, endothelial dysfunction, inflammation, and platelet function and coagulation. Q = Question (see “Methods” section). IFE = immediately following exposure to HTP. *Duration = lowest duration deviation observed or longest duration no deviation observed. †Sufficient duration = HTP trial duration equal to or longer than evidenced time to deviation upon cigarette cessation.- no data available/evidence found.

**Figure 2.**
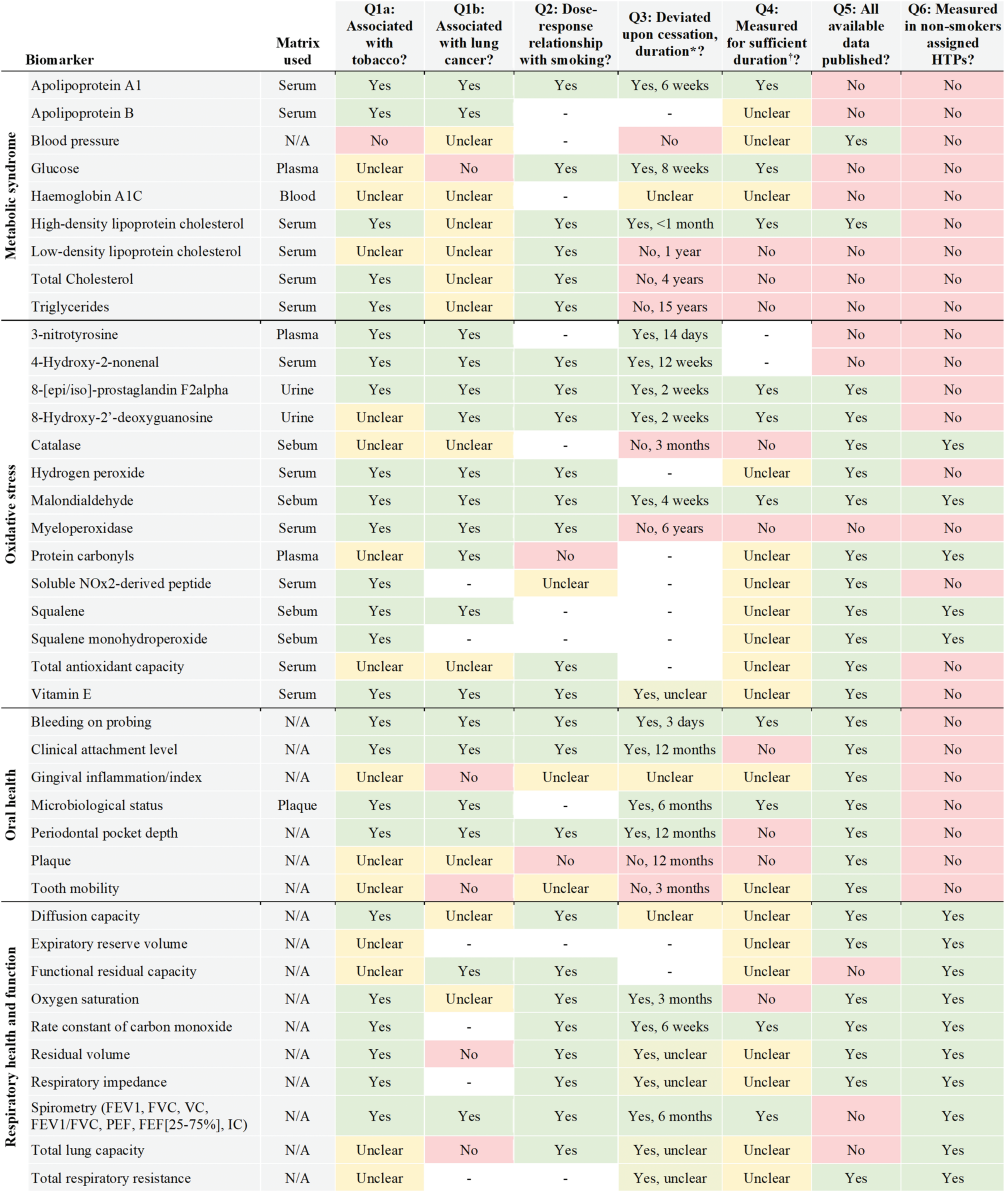
Summary of evaluation of biomarkers relating to metabolic syndrome, oxidative stress, oral health, and respiratory health and function. Q = Question (see “Methods” section). IFE = immediately following exposure to HTP. *Duration = lowest duration deviation observed or longest duration no deviation observed. †Sufficient duration = HTP trial duration equal to or longer than evidenced time to deviation upon cigarette cessation.– no data available/evidence found.

The evidence used to address each of the questions is summarized in Figures 1 and 2. Findings and data used to answer the questions are provided in Supplementary Table S1.

We identified numerous types of biomarkers measured in the HTP clinical trials, namely biomarkers of exposure, endothelial dysfunction, inflammation, platelet function and coagulation, metabolic syndrome, oxidative stress, oral health, and respiratory health and function. The biomarkers were measured in surrogate matrices, mostly urine and blood, with the exception of biomarkers of oral health, respiratory health and function, and those measured in sebum.

The following 22 biomarkers were associated with tobacco use and lung cancer risk, dose-dependently correlated with smoking, and modifiable upon cessation: monohydroxybutenylmercapturic acid, 4-hydroxybutyl-2-mercapturic acid, 2-hydroxyethylmercapturic acid, nicotine, cotinine, nicotine equivalents, total 4-(methylnitrosamino)-1-(3-pyridyl)-1-butanol, endothlin-1, nitric oxide, high sensitivity C-reactive protein, white blood cell count, fibrinogen, soluble p-selectin, apolipoprotein A1, 4-Hydroxy-2-nonenal, 8-[epi/iso]-prostaglandin F2alpha, malondialdehyde, vitamin E, bleeding on probing, clinical attachment level, periodontal pocket depth, and spirometry measures. However, four of these were not measured in an appropriate timeframe based on reversibility (high sensitivity C-reactive protein, soluble p-selectin, clinical attachment level, and periodontal pocket depth), and no results data have been published from the HTP clinical trials on endothelin-1 or 4-Hydroxy-2-nonenal. Only malondialdehyde and spirometry have been measured in non-smokers exposed to HTPs.

#### Association with Tobacco and Lung Cancer (Question 1)

According to existing evidence, most of the biomarkers were significantly associated with tobacco use. Only S-benzyl mercapturic acid and blood pressure were not associated with tobacco use. It was unclear whether there was an association between tobacco use and 14 of the biomarkers due to insignificant or conflicting findings among existing literature.

Thirty-five of the biomarkers were also associated with lung cancer risk. 4-aminobiphenyl, 3-hydroxy-1-methylpropylmercapturic acid, total N-nitrosonornicotine, total 1-hydroxypyrene, glucose, gingival inflammation, tooth mobility, residual volume, and total lung capacity were not associated with lung cancer risk. It was unclear whether there was an association between lung cancer risk and 20 of the biomarkers due to insignificant or conflicting findings among existing literature. We found no evidence on the association with lung cancer for 18 biomarkers.

#### Dose–Response Relationship (Question 2)

Fifty-nine biomarkers were dose-dependently correlated with tobacco exposure. 3-hydroxybenzo[a]pyrene, protein carbonyls, and plaque were not dose-dependently correlated with tobacco exposure. The presence of a dose–response relationship was unclear for 6 of the biomarkers due to insignificant or conflicting findings among existing literature. We found no evidence on a dose–response relationship with smoking for 14 biomarkers.

#### Deviation Following Cessation (Question 3)

The effects of smoking on 54 of the biomarkers deviated from the effects of smoking following cessation. Time to deviation across the biomarkers ranged from 12 h up to 15 years. E-selectin, homocysteine, soluble CD40 ligand, blood pressure, low-density lipoprotein cholesterol, total cholesterol, triglycerides, catalase, myeloperoxidase, plaque, and tooth mobility have not been observed in previous studies to reverse upon cessation. The effects of cessation were unclear for three biomarkers and time to deviation was unclear for six biomarkers due to insignificant results, conflicting findings, or unspecified cessation durations in the existing literature. We found no evidence on the effects of cessation for 14 biomarkers.

#### Data Availability (Question 4)

Data on 58 biomarkers were published from every HTP clinical trial in which they were measured. Twenty-five biomarkers were measured in at least one study that has yet to report the relevant results data. There were exhaled carbon monoxide, carboxyhaemoglobin, albumin, endothelin-1, e-selectin, monocyte chemoattractant protein-1, high sensitivity c-reactive protein, homocysteine, white blood cell count, fibrinogen, plasminogen activator inhibitor-1, platelet count, apolipoprotein A1 and B, glucose, hemoglobin A1c, low-density lipoprotein cholesterol, total cholesterol, triglycerides, 3-nitrotyrosine, 4-Hydroxy-2-nonenal, myeloperoxidase, functional residual capacity, spirometry measures except forced expiratory volume in 1 second (FEV1), and total lung capacity.

No data have been published at all from the HTP trials on endtholin-1, e-selectin, monocyte chemoattractant protein-1, plasminogen activator inhibitor-1, 3-nitrotyrosine, and 4-Hydroxy-2-nonenal.

#### Sufficient Follow-up (Question 5)

The longest follow-up for any biomarker for which results data from an HTP trial were published ranged from immediately following exposure up to 12 months. Based on existing evidence on the effects of cessation, follow-up periods were too short for 13 biomarkers: flow-mediated dilation, high sensitivity C-reactive protein, soluble CD40 ligand, soluble p-selectin, low-density lipoprotein cholesterol, total cholesterol, triglycerides, catalase, myeloperoxidase, clinical attachment level, periodontal pocket depth, plaque, and oxygen saturation. Follow-up periods for N-acetyl-S-(2-carba-moylethyl)cysteine and N-acetyl-S-(2-hydroxy-2-carbamoylethyl)cysteine were borderline.

#### Effects Measured in Non-smokers (Question 6)

Exhaled carbon monoxide, catalase, malondialdehyde, protein carbonyls, squalene, squalene monohydroperoxide, diffusion capacity, expiratory reserve volume, functional residual capacity, oxygen saturation, rate constant of carbon monoxide, residual volume, respiratory impedance, FEV1, forced vital capacity, forced expiratory volume in one second/forced vital capacity, peak expiratory flow, forced expiratory flow at 25–75% of forced vital capacity, total lung capacity, and total respiratory resistance have all been measured in non-smokers who were directly exposed to or used HTPs.

### Effect of HTPs on Most Appropriate Biomarkers

Findings from the HTP clinical trials relating to each of the 16 biomarkers judged to be most appropriate in assessing lung cancer risk are summarized in Table 2. Only studies that were of sufficient duration in line with the evidence on time to reversal or deviation upon cessation were included. The earliest evidenced time to reversal for the three nicotine-related biomarkers was 5.5 days. Findings from 5-day trials were included given the proximity of the timeframes. An overview of the HTP trials included in this synthesis and which of the 16 biomarkers they reported data on is provided in Supplementary Table S2.

**Table 2.** The effects of HTPs on the most appropriate biomarkers for assessing lung cancer risk. (Number of trials, trial durations)

Biomarker		Effect in smokers switched to HTPs	Effect in non-smokers switched to HTPs	Last follow-up difference between HTP and cigarette groups	Last follow-up difference between HTP and cessation groups
Biomarkers of exposure	Monohydroxybutenyl mercapturic acid	Significant decrease (12 trials, 5 days–6 months)^34–45^	*Not measured in any trials that included non-smokers.*	Significantly lower in HTP group (13 trials, 5 days–6 months)^34–46^	Not significantly different (9 trials, 5 days–6 months)^36,38–41,43–45^
	4-Hydroxybutyl-2-mercapturic acid	Significant decrease (1 trial, 5 days)^44^	*Not measured in any trials that included non-smokers.*	Significantly lower in HTP group (1 trial, 5 days)^44^	Not significantly different (1 trial, 5 days)^44^
	2-Hydroxyethyl mercapturic acid	Significant decrease (10 trials, 5 days–6 months)^35–41,43–45^	*Not measured in any trials that included non-smokers.*	Significantly lower in HTP group (11 trials, 5 days–6 months)^35–41,43–46^	Not significantly different (9 trials, 5 days–6 months)^36,38–41,43–46^
	Nicotine	No significant change (7 trials, 5 days–6 months)^37,41,43,47–49^	*Not measured in any trials that included non-smokers.*	Not significantly different (7 trials, 5 days–6 months)^37,41,43,47–49^	Significantly higher in HTP group (4 trials, 5–90 days)^41,43,47,48^
	Cotinine	No significant change (7 trials, 5 days–6 months)^37,41–43,47–49^	*Not measured in any trials that included non-smokers.*	No significant difference (7 trials, 5 days–6 months)^37,41–43,47–49^	Significantly higher in HTP group (4 trials, 5–90 days)^41,43,47,48^
	Nicotine equivalents	No significant change (9 trials, 5 days–6 months)^34,35,37,40–43,45,50^ Significant decrease (4 trials, 5 days–6 months)^36,38,39,44^	*Not measured in any trials that included non-smokers.*	No significant difference (10 trials, 5 days–6 months)^34,35,37,38,40–43,45,50^ Significantly lower in HTP group (3 trials, 5 days)^36,39,44^	Significantly higher in HTP group (8 trials, 5–6 months)^36,38–41,43–45^
	Total 4-(methyl nitrosamino)-1-(3-pyridyl)-1-butanol	Significant decrease (12 trials, 5 days–6 months)^34–40,42–45,50^ No significant change (1 trial, 5 days)^41^	*Not measured in any trials that included non-smokers.*	Significantly lower in HTP group (15 trials, 5 days–12 months)^34–46,50,51^	Not significantly different (7 trials, 5 days–90 days)^39–41,43–46^ Significantly higher in HTP group (2 trials, 5 days–6 months)^36,38^
Biomarkers of potential harm	Nitric oxide	FeNO: increase (significance not reported; 1 trial, 6 months)^38^	*Not measured in any trials that included non-smokers.*	FeNO: significantly higher in HTP group (1 trial, 6 months)^38^	Unclear due to insufficient data reported (1 trial, 6 months)^38^
	White blood cell count	No significant change (3 trials, 90 days–6 months)^34,52,53^ Significant decrease (1 trial, 90 days)^35^ Significant increase (1 trial, 6 months)^38^	*Not measured in any trials that included non-smokers.*	Significantly lower in HTP group (5 trials, 90 days–12 months)^34,35,38,51,52^ Not significantly different (1 trial, 90 days)^53^	Not significantly different (1 trial, 90 days)^52^ Significantly higher in HTP group (1 trial, 90 days)^53^
	Fibrinogen	No significant change (1 trial, 6 months)^49^	*Not measured in any trials that included non-smokers.*	No significant difference (1 trial, 6 months)^49^	*Not measured in any trials of sufficient length with a cessation arm.*
	Apolipoprotein A1	No significant change (1 trials, 6 months)^49^	*Not measured in any trials that included non-smokers.*	Significantly higher in HTP group (1 trial, 6 months)^49^	*Not measured in any trials of sufficient length with a cessation arm.*
	Bleeding on probing	Significant decrease (1 trial, 6 months)^50^	*Not measured in any trials that included non-smokers.*	No significant difference (1 trial, 6 months)^50^	*Not measured in any trials of sufficient length with a cessation arm.*
	8-[epi/iso]-prostaglandin F2alpha	No significant change (3 trials, 90 days)^35,52,53^ Significant decrease (2 trials, 6 months)^34,38^	*Not measured in any trials that included non-smokers.*	Significantly lower in HTP group (4 trials, 90 days–6 months)^34,38,52,53^ No significant difference (2 trials, 90 days–12 months)^35,51^	Not significantly different (2 trials, 90 days)^52,53^
	Malondialdehyde	Significant decrease (1 trial, 1 month)^54^	*Not measured in any trials of sufficient length with a cessation arm.*	Significantly lower in HTP group (1 trial, 1 month)^54^	*Not measured in any trials of sufficient length with a cessation arm.*
	Vitamin E	No significant change (1 trial, single use)^55^	*Not measured in any trials that included non-smokers.*	Significantly higher in HTP group (1 trial, single use)^55^	*Not measured in any trials of sufficient length with a cessation arm.*
	Spirometry	FEV1: no significant change (2 trials, 6 months)^34,38^	*Not measured in any trials of sufficient length with a cessation arm.*	FEV1: no significant difference (2 trials, 6–12 months)^38,51^ Significantly higher in HTP group (1 trial, 6 months)^34^	Unclear due to insufficient data reported (1 trial, 6 months)^38^

HTP = heated tobacco product; N/A = not applicable; FeNO = fractional exhaled nitric oxide; FEV1 = forced expiratory volume in 1 s.

Two studies measured HTP effects in non-smokers, specifically on malondialdehyde and spirometry. However, neither was of sufficient length in relation to the evidence on time to reversal or deviation following cessation. This meant there were no appropriate data indicative of the lung cancer risk in non-smokers exposed to HTPs.

Monohydroxybutenylmercapturic acid, 4-hydroxybutyl-2-mercapturic acid, and 2-hydroxyethyl mercapturic acid improved in smokers who switched to HTPs were less negatively affected by HTPs relative to cigarettes and were not significantly different from smokers who ceased smoking.

Expectedly, nicotine-related biomarkers were largely unchanged compared and remained higher in smokers who switched to HTPs than those who ceased smoking. Fractional exhaled nitric oxide and fibrinogen remained unchanged or significantly worsened in smokers who switched to HTPs, both in comparison to cigarettes and between baseline and last follow-up.

When examining a change between baseline and last follow-up in smokers switched to HTPs: bleeding on probing and malondialdehyde significantly improved; nitric oxide significantly worsened; fibrinogen, apolipoprotein A1, vitamin E and FEV1 remained unchanged; and the effects on total 4-(methylnitrosamino)-1-(3-pyridyl)-1-butanol, white blood cell count and 8-[epi/iso]-prostaglandin F2alpha were unclear due to conflicting findings across studies.

In comparison to continued smoking, switching to HTPs resulted in significant improvement in total 4-(methylnitrosamino)-1-(3-pyridyl)-1-butanol, apolipoprotein A1, malondialdehyde and vitamin E; significant worsening in nitric oxide; no significant difference in fibrinogen and bleeding on probing; and no clear effect across studies on 8-[epi/iso]-prostaglandin F2alpha, white blood cell count and spirometry.

Switching to HTPs caused no significant difference in 8-[epi/iso]-prostaglandin F2alpha compared to cessation. There was no clear effect across studies on total 4-(methylnitrosamino)-1-(3-pyridyl)-1-butanol and white blood cell count. Insufficient data were reported on nitric oxide and spirometry to determine the relative effects of HTPs and cessation in smokers.

## Discussion

Of the 82 biomarkers evaluated, 16 were associated with tobacco use and lung cancer, dose-dependently correlated with smoking, modifiable upon cessation, measured within an appropriate timeframe and have had results data published. Seven of these are biomarkers of exposure and nine biomarkers of potential harm. Of these, just three of the exposure biomarkers were consistently significantly improved in smokers by a switch to HTPs, both between baseline and last follow-up, and compared to continued smoking or cessation. This is arguably to be expected given HTPs emit lower levels of the chemicals these biomarkers measure.^56^ The remaining 13 biomarkers either did not improve or were differently effected by HTPs across studies. Malondialdehyde and spirometry have been measured in non-smokers assigned HTPs, but neither has been measured in trials of sufficient duration based on evidenced timeframes of deviation following cessation.

All but 2/35 of HTP clinical trials measuring biomarkers were switch studies, meaning they compared smokers who switched to HTPs to smokers who continued smoking cigarettes. These studies can only provide evidence on the relative risk of HTPs compared to cigarettes. A reduction in the effects caused by cigarettes may not be sufficient to reduce the risk of disease and death if the levels in HTPs may remain high enough to trigger disease development. This is particularly salient with regards to the biomarkers which worsened in smokers who switched to HTPs, despite appearing improved compared to the cigarette group.

As previously mentioned, should lung cancer manifest in smokers who switch to HTPs, it is difficult to determine which product triggered development due to possible residual effects of smoking. To better ascertain the true absolute lung cancer risk of HTPs, their effects relative to cessation and effects in never smokers must be examined. Yet, there were few studies that conducted these examinations, meaning the absolute risks of HTPs, or indeed whether they are risk-free, remains unknown.

In the absence of epidemiological data on disease incidence, biomarker data are used by HTP manufacturers to substantiate reduced-risk claims.^15–17^ Evaluating the veracity of these claims, notably by regulators and policy makers, is hindered by a lack of proper biomarker evaluation, especially in regard to the use of biomarkers in place of clinical endpoints (i.e. disease manifestation).^57^ Indeed, while some of the biomarkers we evaluated were related to the various physiological changes involved in lung cancer development, and thus indicative of risk, there were no validated surrogate markers that definitively predicted lung cancer manifestation.

Many of the biomarkers measured across the HTP trials have not been repeatedly measured over multiple studies and there is a wealth of unpublished data. This limits the reliability of existing biomarker data as it is unclear whether the biomarkers are predictable and results reproducible. Indeed, we identified conflicting findings across the HTP clinical trials for many of the 16 biomarkers potentially indicative of lung cancer risk.

The tobacco industry has developed simulation models to estimate the risks of lung cancer from HTPs in pre-market settings using aerosol analyses, and *in vitro* and *in vivo* data. Unsurprisingly, the conclusion of these simulations is that HTPs reduce the risk of lung cancer compared to smoking.^58–61^ The findings of our evaluation suggest much of the existing biomarker data may not be appropriate for estimating the lung cancer risk posed by HTPs. Therefore, in addition to the flaws noted by others,^62^ our findings bring into question the accuracy of such simulation models.

### Strengths and Limitations

Expanding upon previous evaluations of biomarkers used to assess novel tobacco and nicotine products, we evaluated the biomarkers used in existing HTP interventional trials and their appropriateness in assessing the lung cancer risk of HTPs. Further, we go beyond existing literature to examine the lung cancer risk of HTPs based on data available for the most appropriate biomarkers identified in our evaluation.

Given the large number of biomarkers to be evaluated, we chose to conduct rapid narrative reviews of relevant literature on each biomarker, as opposed to systematic reviews that could be more comprehensive. There are ideal characteristics of biomarkers that were not assessed in our evaluation, namely specificity, reproducibility, temporality, and analogy. Exploring these characteristics could be worthwhile to further inform future HTP clinical research and evaluation of industry-reduced risk claims based on existing biomarker data.

### Recommendations for Future Research

Evidently, the ultimate recommendation for future research on the lung cancer risk of HTPs is to conduct observational studies, both cross-sectional and longitudinal, measuring lung cancer incidence amongst HTP users. However, as previously mentioned, this may not yet be feasible due to the long latency period and residual effects of smoking. Thus, in lieu of these observational studies, interventional clinical trials measuring risk biomarkers will continue to be of utmost importance.

Investigators assessing the reduced risk potential of HTPs should carefully consider and clearly justify biomarker selection and study design, ensuring both aspects align with existing evidence.

Numerous biomarkers known to be significantly associated with lung cancer risk have not thus far been measured in individuals exposed to HTPs, such as protein and antibody panels,^32,63^ urinary and circulating metabolites,^64,65^ and other inflammation^66^ and oxidative stress markers.^28^ Given the prevalence and seriousness of the disease, future clinical studies should prioritize biomarkers associated with lung cancer. Moreover, as some biomarkers are more informative and better indicators of disease risk when measured in local target tissues,^24^ measuring biomarkers in lung-specific matrices should be explored.

Selective reporting bias can compromise trial validity and skew the evidence base. This is particularly relevant in the HTP clinical evidence base, which is dominated by industry studies^56^ and still relatively limited. Researchers should endeavor to publish results data on all biomarkers measured via one of the many channels available, for example, trial registrations and journal publications.

There is evidence of HTP use among never smokers.^67–69^ We cannot recommend the inclusion of never smokers in interventional clinical trials on HTPs given the obvious ethical issues around giving participants a harmful intervention. Nonetheless, data from never smokers is of great value in determining the absolute risks of HTPs. In order to obtain such data, future research could use observational designs to investigate biomarkers or disease outcomes in never smokers who already use HTPs.

## Conclusion

In conclusion, despite a wide array of biomarkers being measured, few could be considered appropriate for assessing the lung cancer risk of HTPs. Furthermore, the findings on the most appropriate biomarkers largely showed no benefit to smokers who switched to HTPs. The HTP clinical trials published to date are of short duration (all 12 months or less) and the findings across the trials were often conflicting, providing no clear indication as to the lung cancer risk of HTPs, neither in terms of absolute risk nor relative to continued smoking or cessation. Future clinical research on HTPs should incorporate observational designs measuring more established and specific biomarkers of lung cancer.

## Supplementary Material

A Contributorship Form detailing each author’s specific involvement with this content, as well as any supplementary data, are available online at https://academic.oup.com/ntr.

ntad081_suppl_Supplementary_Material

## Data Availability

The data underlying this article are available in the article and in its online Supplementary Material.
