# Supplementary material for: What Can Current Biomarker Data Tell Us About the Risks of Lung Cancer Posed by Heated Tobacco Products?
Source: Nicotine Tob Res. 2023 May 21;26(3):270–80. doi: 10.1093/ntr/ntad081 (PMC10882439; doi:10.1093/ntr/ntad081)
Supplement: ntad081_suppl_Supplementary_Material [file ntad081_suppl_supplementary_material.docx]

# Supplementary Table 1. Summary of findings from narrative review on each biomarker.

|  | **Biomarker** | **Association with tobacco** | **Association with lung cancer (LC)** | **Dose-response relationship with smoking cigarettes** | **Deviation following cigarette smoking cessation** | **Longest published follow-up in HTP trials** | **Number of studies (reported data/ measured biomarker)** |
| --- | --- | --- | --- | --- | --- | --- | --- |
| **Exposure** | Monohydroxybutenylmercapturic acid | Associated with smoking (higher in smokers than non-smokers).^1-5^ | Significantly higher in LC patients than healthy controls and thus associated with LC risk.^6^ | Significantly, but moderately, associated with cigarettes-per-day^6^ and nicotine equivalents. ^3^ | Significantly decreases after 3 days’^7^ and 5.5 days’ cessation.^8^ | 6 months | 13/13 |
|  | 2-aminonaphthalene | Associated with smoking (higher in smokers than non-smokers). ^9-11^ | While related to overall cancer risk ^12^and risk of bladder cancer,^13^*we found no direct evidence of an association with LC*. | Significantly correlated with exhaled CO, but not cotinine, nicotine equivalents or cigarettes-per-day. ^10^ | Significantly reduced in quitters after 5.5 days. ^8^ | 6 months | 12/12 |
|  | 4-aminobiphenyl | Associated with smoking (higher in smokers than non-smokers). ^2,3,10,14^ | Related to overall cancer risk ^15^ and risk of bladder cancer.^16^ Levels of its haemoglobin adducts are not significantly associated with LC risk.^15,17^ | Dose-response effect exhibited with smoking.^10,14^ | Significantly reduced in quitters after 5.5 days. ^8^ | 6 months | 12/12 |
|  | 3-hydroxypropylmercapturic acid | Associated with smoking (higher in smokers than non-smokers).^1-3,5,18^ | Significantly higher in LC patients than healthy controls and thus associated with LC risk.^6,19^  However, not significantly different between never smoker LC patients and healthy controls.^20^ | Strong linear relationship with nicotine equivalents. ^3^ Significantly moderately associated with cigarettes-per-day. ^6^ | Significantly decreases after 3 days’^7^and 5.5 days’ cessation. ^8^ | 6 months | 13/13 |
|  | N-acetyl-S-(2-carba-moylethyl)cysteine | Significantly higher in smokers than non-smokers.^1,21^ | *No evidence on an association with LC found.* | Significantly positively correlated with cotinine.^22^ | Significantly reduced in quitters after 5.5 days. ^8^ | 5 days | 2/2 |
|  | N-acetyl-S-(2-hydroxy-2-carbamoylethyl)cysteine | Significantly higher in smokers than non-smokers.^1,21^ | *No evidence on an association with LC found.* | Significantly positively correlated with cotinine.^22^ | Significantly reduced in quitters after 5.5 days. ^8^ | 5 days | 2/2 |
|  | S-phenylmercapturic acid | Associated with smoking (higher in smokers than non-smokers).^1,2,5,23^ | One study found it was significantly higher in LC patients than healthy controls and thus associated with LC risk.^6^ However, another study suggests that while it is related to cancer risk, it is not specifically associated with LC risk.^20,24^ | Significantly associated with cigarettes-per-day ^6^ and cotinine.^22^ | Significantly decreases after 3 days’^7^and 5.5 days’ cessation. ^8^ | 6 months | 13/13 |
|  | 3-hydroxybenzo[a]pyrene | Associated with smoking (higher in smokers than non-smokers).^23,25^ | Measures exposure to benzo[a]pyrene, which is associated with lung cancer development,^26^ but *we found no direct evidence of an association with LC*. | Not significantly correlated with cotinine.^27^ | Significantly reduced in quitters after 5.5 days. ^8^ | 6 months | 9/9 |
|  | 3-hydroxy-1-methylpropylmercapturic acid | Associated with smoking (higher in smokers than non-smokers).^1,21^ | No significant difference between LC patients and healthy controls (non-smokers).^20^ | Significantly associated with cigarettes-per-day.^28^ | Significantly reduced in quitters after 5.5 days. ^8^ | 6 months | 11/11 |
|  | 4-hydroxybutyl-2-mercapturic acid | Associated with smoking (higher in smokers than non-smokers). ^2,5^ | Significantly higher in LC patients than healthy controls and thus associated with LC risk. ^6^ | Significantly moderately associated with cigarettes-per-day. ^6^ | Significantly decreases after 3 days’ cessation. ^7^ | 5 days | 1/1 |
|  | 2-hydroxyethylmercapturic acid | Associated with smoking (higher in smokers than non-smokers).^1,2,5,21^ | Significantly higher in LC patients than healthy controls and thus associated with LC risk. ^6^ | Significantly associated with cigarettes-per-day. ^6^ | Significantly decreases after 3 days’^7^and 5.5 days’ cessation. ^8^ | 6 months | 11/11 |
|  | Urine mutagenicity | Associated with smoking.^13,14,29^ | Proposed as a marker of LC as an indicator of genetic damage and overall risk of cancer,^30^ but *we found no direct evidence of an association with LC*. | Dose-response effect exhibited with smoking.^13,14,31^ | Significantly reduced in quitters compared to continued smokers (20 cigs/day) after 5.5 days. ^8^ | 3 months | 6/6 |
|  | Nicotine | Associated with smoking.^5,14,25,31^  Significantly higher in smokers than non-smokers.^1^ | Nicotine plays an important role in lung cancer development, including promoting development and reducing treatment effectiveness.^32^  Nicotine promotes tumour growth and metastasis.^33^ | Dose-response effect exhibited with smoking.^14^ | Significantly reduced in quitters after 5.5 days. ^8^ | 6 months | 13/13 |
|  | Cotinine | Associated with smoking.^5,14,25^  Significantly higher in smokers than non-smokers. ^1,3^ | Dose-dependently associated with LC risk.^5,25^ | Positively associated with cigarettes-per-day.^6,25,34^ | Significantly reduced in quitters after 5.5 days. ^8^ | 6 months | 10/10 |
|  | Nicotine equivalents | Associated with smoking.^5,25,31^  Higher in smokers than non-smokers. ^2^ | Significantly higher in LC patients than healthy controls.^35^  Significantly associated with LC risk.^19^ | Strongly correlated with multiple tobacco exposure biomarkers.^25^ | Significantly reduced in quitters after 5.5 days. ^8^ | 6 months | 14/14 |
|  | Total 4-(methylnitrosamino)-1-(3-pyridyl)-1-butanol | Associated with smoking (higher in smokers than non-smokers).^2,3,5,14,25^ | Significantly dose-dependently (positively) associated with LC risk.^5,25^ | Positively associated with cotinine, nicotine ^3,25^ and cigarettes-per-day.^6,34^  Dose-response relationship with smoking. ^14^ | 34% remains for up to 1 week following cessation.^25^  Significantly decreases after 3 days’^7^and 5.5 days’ cessation. ^8^ | 12 months | 15/15 |
|  | Total N-nitrosonornicotine | Associated with smoking (higher in smokers than non-smokers).^2,5,25^ | Not associated with lung cancer. ^25^ | Positively associated with cigarettes-per-day and cotinine. ^25^ | Significantly reduced in quitters after 5.5 days. ^8^ | 6 months | 14/14 |
|  | o-toluidine | Associated with smoking (higher in smokers than non-smokers).^10,11,21^ | Induces genetic damage^36^ and associated with bladder cancer, ^16^ but *we found no direct evidence of an association with LC*. | Significantly correlated with nicotine equivalents but not exhaled CO, cotinine or cigarettes per day.^10^ | Significantly reduced in quitters after 5.5 days. ^8^ | 6 months | 12/12 |
|  | Cytochrome P450 2A6 activity | Involved in nicotine metabolism and can modify internal smoking dose.^37^  One study found it was significantly associated with cigarette consumption,^38^ while another found smoking had no effect on activity.^39^ | Significantly positively associated with lung cancer risk.^19,37,40^ | Significantly positively associated with cigarette consumption*.*^38,41^ | While CYP2A6 plays an important role in smoking behaviour and cessation,^40^ *we found no evidence on reversibility.* | 6 months | 8/8 |
|  | total 1-hydroxypyrene | Associated with smoking (higher in smokers than non-smokers).^2,3,5,25^ | Not significantly different between LC patients and healthy controls.^42,43^ | Correlated with cigarettes-per-day.^34,44^ | Significantly decreases after 3 days’^7^and 5.5 days’ cessation. ^8^ | 6 months | 13/13 |
|  | 2-cyanoethylmercapturic acid | Significantly higher in smokers than non-smokers.^1,21^ | *No evidence on an association with LC found.* | Significantly correlated with ISO tar yield, cigarettes-per-day and nicotine equivalents.^45^ | Significantly reduced in quitters after 5.5 days. ^8^ | 6 months | 14/14 |
|  | 1-aminonaphthalene | Associated with smoking (higher in smokers than non-smokers). ^9,21^ | *No evidence on an association with LC found.* | *No evidence on a dose-response relationship found.* | *No evidence on reversibility found.* | 3 months | 8/8 |
|  | Exhaled Carbon monoxide | Associated with smoking (higher in smokers than non-smokers.^2,14,25,31^ | *No evidence on an association with LC found.* | Has dose-response relationship with smoking.^14,25^ | Significantly reduced in quitters after 5.5 days. ^8^  Not significantly different between smokers and quitters after 1 day, but significantly lower in quitters after 8 days.^46^ | 6 months | 21/26 |
|  | Carboxyhemoglobin | Associated with smoking (higher in smokers than in non-smokers).^2,3,14,25^ | Increase may be related to worsened prognosis^47^ and recurrence.^48^ | Has dose-response relationship with smoking.^3,14,25^ | Significantly reduced in quitters after 5.5 days. ^8^  Significantly reduced after 12 hours’ cessation.^49^ | 12 months | 13/14 |
|  | N-(2-cyanoethyl)valine haemoglobin adducts | Associated with smoking (higher in smokers than non-smokers).^50,51^ | *No evidence on an association with LC found.* | Significantly correlated with smoking dose and cotinine.^50^ | *No evidence on reversibility found.* | 6 months | 1/1 |
|  | S-benzyl mercapturic acid | Not significantly different between smokers and non-smokers. ^1,52,53^ | *No evidence on an association with LC found.* | *No evidence on a dose-response relationship found.* | *No evidence on reversibility found.* | 3 months | 4/4 |
| **Endothelial Dysfunction** | Albumin | Significantly lower in smokers than non-smokers.^54^ | No significant associations between albumin and LC.^55,56^  Inverse association between LC risk and albumin observed in Chinese^57^ and African Americans ^58^ suggesting association may vary by race. | Strongly inversely associated with smoking.^54,59,60^ | Significantly increased in quitters after 6 months’ cessation and normalised to never smoker levels after 9 months.^61^  Not significantly associated with time since cessation, but significantly different between former and current smokers.^54^  No significant difference between quitters and continued smokers after 26 weeks’ cessation.^62^  Levels normalise to never smoker levels after 5 years’ cessation,^59^ but could take >10 years to normalise.^60^ | 6 months | 1/2 |
|  | Endothelin-1 | Significantly higher in smokers than non-smokers.^63-65^ | ET-1 has important role in growth, proliferation and maintenance of lung tumour cells.^66-68^  Significantly higher in patients with NSCLC than healthy controls.^69,70^ | Significantly positively associated with smoking.^64^ | Significantly reduced compared to baseline following 12 weeks’ cessation.^71^ | N/A | 0/1 |
|  | E-selectin | Significantly higher in current smokers than non-smokers^72-75^, but association  not consistent across all studies.^76,77^ | Significantly elevated in LC patients compared to healthy controls.^78-80^ | Positively associated with pack years in women, but not men.^74^ | Not statistically significantly different between smokers and quitters after 1 year.^81^ | N/A | 0/1 |
|  | Flow mediated dilation | There is strong association between FMD and pack years smoked.^82,83^ | *No evidence on an association with LC found.* | Smoking impairs FMD in a dose-dependent manner ^82,83^ | Significant improvement of FMD following 1 year cessation. ^82,84^ | 1 month | 2/2 |
|  | Nitric oxide | Exhaled NO^85-88^ and NO bioavailability^89-91^ significantly lower in smokers than non-smokers.  Oxidised NO^92,93^ and plasma NO^94^ significantly higher in smokers than non-smokers. | Significantly higher in smokers than in healthy subjects.^95-97^  Plays complex role in cancer development: reduced levels can increase vascularity and protect cells, including cancerous cells, from apoptosis, and may promote tumour growth.^95,98^ | Significant negative correlation between exhaled NO and cigarettes per day.^86,88^ | Significantly improved following 1 week cessation and normalises to never smoker levels after 4-8 weeks’ cessation.^85,87^ | 6 months | 2/2 |
|  | Soluble intercellular adhesion molecule-1 | Significantly higher in current smokers than non-smokers.^72,73,99-101^ | Significantly higher in LC patients than healthy controls.^102^  Significantly positively correlated with tumour size.^103^  Not significantly associated with LC risk in smokers or non-smokers.^104^ | Dose-dependently associated with daily cigarette consumption, plasma cotinine, and exhaled carbon monoxide.^72,99^ | Significantly different between smokers and quitters after 1 year.^81^  Significantly declined in smokers after 4 weeks-3 months’ cessation.^100,105-107^ | 12 months | 6/6 |
| **Inflammation** | Monocyte chemoattractant protein-1 (CCL2) | Significantly increased in smokers’ BAL compared to BAL of non-smokers.^108^ | Involved in proliferation, migration and survival of lung tumour cells.^109,110^  Associated with increased risk of LC in Shanghai female never smokers.^111^  CCL2 gene polymorphism associated with LC risk.^112^  Not significantly associated with LC risk in smokers or non-smokers.^104^ | *No evidence on a dose-response relationship found.* | *No evidence on reversibility found.* | N/A | 0/1 |
|  | High sensitivity C- reactive protein | Strong association with smoking. ^54,60,72,113^ | Significantly, independently, positively associated with LC risk.^114-119^ | Strong, positive, independent, and dose–response relationship with smoking.^54,113^ | Several studies have found no significant change following cessation of up to a year.  No significant change after 4 years’ cessation.^120^  May take 5-20 years to normalise to never smoker levels following cessation.^54,72,121,122^  Significantly negatively associated with time since cessation.^54^ | 6 months | 4/6 |
|  | Homocysteine | Significantly higher in current smokers than never smokers.^101,113,122-124^ | Higher in LC patients than healthy controls.^57^  Elevated in blood circulation is likely risk factor for carcinogenesis.^125^  Not significantly different between smokers diagnosed LC and healthy smokers.^126^ | Strong, positive, independent, and dose–response relationship with smoking.^113^ | No different between quitters and smokers after 10-11 weeks.^127^  Could take 10+ years to normalise to never-smoker levels.^122^ | 6 months | 4/6 |
|  | White blood cell count | Significantly increases with smoking.^60,72^ ^101,128^ | Positively associated with LC risk.^129,130^ | Significantly positively associated with increased smoking intensity (WBC increases as cc/day increases).^54^ | Significantly negatively associated with time since cessation.^54^  Normalisation typically after 1 year of cessation, but may 5+ years.^54,60,61,131,132^  Some sub-types can significantly decrease within 24hrs of abstinence^131^, but whole WBC reduction likely observable after 2 weeks’ cessation.^133^ | 12 months | 7/8 |
| **Metabolic syndrome** | Apolipoprotein A1 | Apo A is decreased in smokers compared to non-smokers.^134-136^ | Negatively associated with LC risk.^137,138^ | Significant dose-response effect. ^135,136^ | Increases following 6-12 weeks’ cessation, though this change is not always significant across studies.^134^ | 6 months | 3/4 |
|  | Apolipoprotein B | Increased in smokers compared to non-smokers.^134^ | Positively associated with LC risk in men.^137^ ^138^ | *No evidence on a dose-response relationship found.* | A 2022 systematic review found only one study presenting data on Apo B levels following cessation, showing Apo B decreased following cessation, but this study was one of the HTP clinical trials.^134^ | 6 months | 3/4 |
|  | Blood pressure | Smoking causes a short-lived temporary increase,^139^ but the chronic effects of smoking on BP is either small and inconsistent or non-existent, with smokers often exhibiting similar or lower BP to non-smokers.^73,140-146^ | May be related to LC incidence in men^147^ and high variability in systolic BP may be associated with LC risk,^148^ an independent, direct effect on LC risk has not been confirmed.^149,150^ | *No evidence on a dose-response relationship found.* | Changes following cessation are nonsignificant and/or inconsistent across different race and sex groups.^143,146,151-153^ | 6 months | 10/10 |
|  | Glucose | Some studies observed a significant increase with smoking,^154-156^ others observed no significant difference between smokers and non-smokers.^157-159^ | Not strongly or significantly associated with LC risk.^150,160-162^  High variability in fasting blood glucose associated with increased LC.^148^ | Positively associated with number of pack-years.^163^ | Significantly increases following 8 weeks’ cessation, remaining higher than never smokers,^164,165^ and may not normalise to never smoker levels until after 15+ years of cessation.^159^ | 3 months | 3/4 |
|  | Haemoglobin A1C | Not significantly different between smokers and never smokers.^73^ ^157^  Significantly higher in smokers than non-smokers.^166,167^ | Some studies found significant positive association with LC risk,^161,168,169^others found no significant association, ^150,170^ one found the association was only significant if levels were moderately elevated but not highly elevated,^171^and one study found the association was significant amongst smokers but not never smokers.^172^ | *No evidence on a dose-response relationship found.* | Increases following cessation for up to 3 years, but not significantly different from active smokers. Normalises to non-smoker levels after around 10 years of cessation.^167^ | 6 months | 4/5 |
|  | High-density lipoprotein cholesterol | Significantly lower in smokers than non-smokers.^73,135,144,157,173,174^ | Significantly lower in LC patients than healthy controls.^175^  Negatively associated with LC risk,^150,176-179^ though no significant associations found in one cohort of Chinese males.^180^ | Significant dose-response effect.^135,181^ | Significantly increases in quitters.^182,183^ This can be observed in <1 month.^184,185^  Levels in quitters can normalise to non-smoker levels after 1 year.^81,144,181^ | 12 months | 6/6 |
|  | Low-density lipoprotein cholesterol | Some studies observed no significant difference between smokers and non-smokers,^73,144,173^ while other studies found LDL-c is significantly higher in smokers.^88,135,157,186-188^  One study found the difference was only significant between smokers who smoked >30 pack-years and never smokers.^189^ | Lower in LC patients but only significantly so in women.^175^  A Mendelian randomisation study and prospective cohort study found inverse association with LC risk.^180,190^  A Mendelian randomisation study and case-control study found no significant association with LC risk. Hao, 2018 #250}^150^ | Significant dose-response effect. ^135,187^ | No significant change and not significantly different from continued smokers following cessation ^182^{Gepner, 2011 #255, not even after 1 year.^81^{Mandraffino, 2017 #218} | 6 months | 4/6 |
|  | Total Cholesterol | TC significantly higher in smokers than non-smokers.^88,135,174,186-189^ | Some studies have observed an inverse association with LC risk,^161,162,177,179^ one study found the association was U-shaped, ^180^ and others found there was no significant association.^150,176^ | Significant dose-response effect.^135,191^ | Multiple studies observed no significant change and not significantly different from continued smokers following cessation, even after 1-4 years. ^81,173,182^ | 3 months | 3/4 |
|  | Triglycerides | Significantly higher in smokers than non-smokers.^73,88,135,157,173,174,186-189^ | Some studies have observed a positive association with LC risk,^161,176,179^one study found the association was U-shaped,^180^ and one study found there was no significant association.^150^ | Significant dose-response effect. ^135^ | No significant improvement and not significantly different from continued smokers following cessation, ^81,182^with levels remaining significantly higher in quitters of 15 years compared to never smokers.^189^ | 3 months | 3/4 |
| **Oxidative stress** | 3-nitrotyrosine | Significantly higher in smokers than non-smokers.^192-197^  One cross-sectional study found levels were consistently lower in smokers and former smokers.^198^ | Significant difference in serum levels between LC patients and healthy controls.^199^  Increased in the tumour relative to the tumour-free region in LC patients.^95^  Significantly increased in human LC tissues and sera compared to controls.^200^ | *No evidence on a dose-response relationship found.* | Numbers of nitrotyrosine positive cells in sputum of smokers remained unchanged after smoking cessation for 3 months.^201^  Intraplatelet nitrotyrosine levels significantly decreased in smokers following 14 days’ cessation.^202^ | N/A | 0/1 |
|  | 4-Hydroxy-2-nonenal | Significantly positively associated with smoking.^203^ | Significantly higher in LC patients than healthy controls. ^175,204^ | Significantly positively associated with smoking.^203^ | Significantly reduced in smokers after 12 weeks’ cessation.^205^ | N/A | 0/2 |
|  | 8-[epi/iso]-prostaglandin F2alpha | Several studies have reported an increased level of 8-iso-PGF2a and 8-epi-PGF2a in smokers compared to non-smokers. ^72,206^ | Significantly higher in LC patients than healthy controls and related to advanced disease.^207^  Significantly associated with LC in former and current smokers. ^206^ | Significantly associated with number of cigarettes smoked per day^208^ and smoking duration.^209^ | Significantly reduced in quitters compared to smokers after 2 weeks’ cessation^202,208^ and approaches normal levels after ~4 weeks cessation.^210^ | 12 months | 10/10 |
|  | 8-Hydroxy-2’-deoxyguanosine | Significantly higher in smokers than non-smokers,^30,194,211-213^though some studies observed no significant difference between smokers and non-smokers. ^30,214^ | Significantly higher in LC patients compared to healthy controls.^215-217^  Increase associated with increased LC risk in never and former smokers.^218,219^ | Positively correlated with cigarettes-per-day and smoking duration.^213,220^ | Significantly reduced in quitters 4 weeks’ cessation.^221,222^ Although, one study observed significant reduction after 2 weeks ^202^and another found levels were non-significantly reduced after 8 weeks.^223^ | 3 months | 1/1 |
|  | Catalase | Some studies found it is significantly lower in smokers than non-smokers ^94,224-226^, while others observed no significant difference between smokers and non-smokers^227-229^. One study found levels were elevated in elderly chronic smokers^230^. | Significantly decreased in tumours compared to tumour-free airway tissues.^231^ | *No evidence on a dose-response relationship found.* | No significant change and not significantly different from continued smokers following up to 3 months’ cessation.^232,233^ | IFE | 1/1 |
|  | Hydrogen peroxide (H_2_O_2_) | Smoking causes influx of WBC which release H2O2 in relatively large amounts compared to non-smokers.^234-238^ | Production higher in LC tissue than pulmonary parenchyma and elevated generation in tumour tissue associated with clinical progression of tumour's stage.^239^  H2O2 contributes to emphysema development^240^ and can damage DNA, which are associated with cancer development, but H2O2 can also have anti-cancer properties.^241^ | There is a positive, significant association between pack-years of smoking and the spontaneous secretion of H2O2.^235^ | *No evidence on reversibility found.* | IFE | 1/1 |
|  | Malondialdehyde | Significantly higher in smokers compared to non-smokers. ^72,242-244^ | Significantly higher in LC patients than healthy controls. ^215,243,245,246^ | Most data suggest there is a positive dose-response relationship.^242^ | Not significantly different following 2 weeks’ cessation.^247^  Significantly reduced and normalised to non-smoker levels after 4 weeks’ cessation.^232,248^  Inversely correlated with smoking cessation duration in LC patients.^246^ | 1 month | 2/2 |
|  | Myeloperoxidase | Smokers have significantly higher levels than non-smokers.^249-251^ | Significantly higher in LC patients than healthy controls, regardless of smoking status.^252^  Gene polymorphism is associated with reduced MPO levels and reduced LC risk.^253^ | Strong independent association with cigarettes-per-day and pack-years.^254^ | Significantly negatively correlated with cessation duration, but not significantly different between quitters and smokers after 6 years’ cessation^254,255^. | 6 months | 2/3 |
|  | Protein carbonyls | Significantly higher in smokers compared to non-smokers.^256-258^  One small study observed no significant difference between smokers and non-smokers.^259^ | Elevated in LC patients compared to heathy controls. ^258^ | No significant differences in carbonyl levels by cotinine levels, pack-years, pack/day or smoking duration.^260^ | *No evidence on reversibility found.* | IFE | 1/1 |
|  | Soluble NOx2-derived peptide | Significantly increased by smoking.^91^  Significantly higher in smokers than non-smokers.^261^  Significantly associated with smoking history^262^and cotinine.^263^ | *No evidence on an association with LC found.* | Significantly correlated with cotinine in passively exposed children.^263^  Not significantly associated with cigarettes-per-day or duration of smoking.^262^ | *No evidence on reversibility found.* | IFE | 1/1 |
|  | Squalene | Exposure to cigarette smoke amplifies squalene oxidation.^264^ Lower in female smokers compared to non-smokers.^265^ | Squalene synthase promotes the invasion and migration of LC cell lines.^266^  Squalene epoxidase (squalene oxidising enzyme) gene is highly expressed in squamous LC tissues and may be closely related to carcinoma development.^267^ | *No evidence on a dose-response relationship found.* | *No evidence on reversibility found.* | IFE | 1/1 |
|  | Squalene monohydroperoxide | Exposure to cigarette smoke amplifies production.^264^ Higher in female smokers than non-smokers.^265^ | *No evidence on an association with LC found.* | *No evidence on a dose-response relationship found.* | *No evidence on reversibility found.* | IFE | 1/1 |
|  | Total antioxidant capacity | Some studies found it is significantly higher in smokers,^268-272^some found it is significantly lower in smokers,^273-277^and others observed no difference between smokers and non-smokers.^278,279^ | Significantly lower in LC patients than healthy controls, ^42,280-282^but this may only be the case amongst non-smokers not smokers.^42,281^  Individuals with low urinary TAC and high oxidative stress have significantly higher LC risk than those with high TAC and low oxidative stress.^42^ | Significant inverse relationship with cigarettes-per-day and smoking duration.^279^ | *No evidence on reversibility found.* | 3 months | 1/1 |
|  | Vitamin E | Significantly lower levels in smokers than non-smokers.^94,194,268,283-287^ | Higher vitamin E status is related in a dose-dependent manner to decreased LC incidence.^288^  Significantly lower in LC patients compared to healthy controls,^175,215^though this inverse association may only be significant in non-smokers.^289^ | There is dose-response relationship with intensity/longevity of smoking ^94^. | No significant change with up to 3 months’ cessation.^233,290^  Levels increase linearly with increasing time since cessation^286^and may normalise with longer periods of cessation. ^287^ | IFE | 1/1 |
| **Oral health** | Bleeding on probing | Some studies observed no significant association with smoking,^291-293^ but most studies found it was significantly lower in smokers than non-smokers.^294-299^  ^300-302^ | Significantly associated with risk of developing LC.^303^ | Smoking causes a strong, chronic, and dose-dependent suppressive effect.^300^ | Significantly increased in smokers who quit for 3 days^304^, 4-6 weeks^305^ and 3 months.^291^  Two studies observed no significant difference between smokers and those who quit for 12 months.^306,307^  One study found current smokers have significantly less gingival bleeding than former smokers who had been abstinent for >1 year.^295^ | 6 months | 1/1 |
|  | Clinical attachment level | Loss of attachment significantly higher in smokers than non-smokers.^294,301,302,308-312^  Significantly associated with smoking.^313^ | Significantly associated with risk of developing LC.^303^  Increased severity in loss significantly increases risk of LC.^314^ | Significantly associated with pack years.^308^  Positive correlation and dose-response relationship with smoking.^294,295,315^ | Loss of attachment significantly higher in quitters following 12 months’ cessation than smokers. ^306^  After 12-24 months, mean CAL gain^316,317^ and reduction in proportion of sites with CAL ≥ 3 mm^316^ significantly higher in quitters compared to smokers.  Mean CAL and percentage of sites with CAL≥5mm lower in ex-smokers compared to smokers.^301,302^ | 6 months | 1/1 |
|  | Gingival inflammation/index | Some studies observed no significant difference between smokers and non-smokers^318-321^ and no significant association with smoking status.^322^  Other studies found a significant association^323^, finding it was significantly lower in smokers than non-smokers. ^296,297,324,325^ | No significant difference between cancer patients and healthy controls.^326^  Not significantly associated with LC risk.^327^ | Significantly lower in heavy smokers than light and moderate smokers,^321^ but some studies have found no association with smoking.^318-320,322^ | Not statistically different between non-smokers, smokers and quitters between baseline and 12 months.^328^  Former/non-smokers had significantly greater GI than smokers.^329^  Lower in ex-smokers and non-smokers compared to smokers.^330^ | 6 months | 1/1 |
|  | Microbiological status | Higher prevalence of some periodontal bacteria in smokers than non-smokers.^294,331-333^  Occurrence, relative frequency or proportion do not differ between smokers and non-smokers.^294,296,333-336^ | Multiple oral bacteria, including orange-complex pathogens, Spirochaetia, Bacteroidetes, Bacilli class and Lactobacillales order, have been shown to be associated with LC risk.^337-343^ | *No evidence on a dose-response relationship found.* | Significantly different between smokers and quitters after 6 months and 12 months.^344^  Former smokers exhibit reduced prevalence and proportions of some bacteria compared to smokers. ^331,332^ | 6 months | 1/1 |
|  | Periodontal pocket depth | Depth and number of sites with greater depths significantly higher in smokers than non-smokers.^294,308,310,311,345^  Significantly associated with smoking.^313^ | Significantly associated with risk of developing LC.^303^ | Positively associated with pack years^308,345^, years of smoking exposure^345^, and amount of smoking.^291^  Positive correlation and dose-response relationship with smoking.^315^ | Continues to increase in quitters after 3 months^291^ and depths remain not significantly different between smokers and those who quit for 12 months.^306^  After 12-24 months cessation, reduction in probing depths is significantly greater in quitters than smokers^306,316^ and depths are significantly reduced in quitters compared to smokers. ^317^ | 6 months | 1/1 |
|  | Plaque | Some studies found it was significantly higher in smokers than non-smokers ^294,301,302,315,330,346^ and significantly associated with smoking.^323,347^  Other studies observed no significant difference between smokers and non-smokers^295,296,318,319,325,328,348,349^ and no significant association with smoking status. ^322^ | No significant difference in between cancer patients and healthy controls.^326^  It has been suggested that the evidenced association between periodontitis and LC may be in part due to aspiration of plaque which contains respiratory pathogens.^327,350^ | Not statistically significantly different based on varying CPD.^348^ | No significant difference between quitters and current smokers, even after 12+ months of cessation.^295,306,328,344^  One study found ex- and non-smokers had significantly greater plaque than smokers^329^, while others found plaque was lower in ex- and non-smokers compared to smokers.^301,302^ | 6 months | 1/1 |
|  | Tooth mobility | Some studies found it is significantly higher in smokers than non-smokers^351-356^, while others observed no significant difference.^293,320,357,358^ | No association between periodontal disease characterised by tooth mobility and LC when other risk factors, including smoking status, are factored in.^359^ | Significantly positively associated with duration of smoking,^360^ but some studies have found no correlated association with smoking.^293,320,357,358^ | No change following aided cessation (Nicorette) for 1-3 months.^361^  Not significantly different between former and current smokers.^293^ | 6 months | 1/1 |
| **Platelet function & coagulation** | 11-dehydrothromboxane B2 | Significantly higher in smokers than non-smokers.^362^ | Higher in NSCLC tissues compared to normal controls.^363,364^ | *No evidence on a dose-response relationship found.* | Significant reductions are observed following as little as 3 days’ cessation.^362^ | 12 months | 10/10 |
|  | Fibrinogen | Several studies demonstrate a strong, independent, positive association with smoking.^60,72,101,113,144,188^ | Significantly associated with LC risk.^365-367^ | Significantly positively associated with cigarettes-per-day^54^demonstrating a dose–response relationship with smoking.^113^ | Significantly negatively associated with time since cessation.^54^  Significantly reduced in quitters after 6 weeks^368^ and 1 year.^144^  Not significantly different from never smoker levels after 5 years’ cessation,^54^ but could take up to 10 years to normalise.^60^ | 6 months | 4/6 |
|  | Plasminogen activator inhibitor-1 | Significantly higher in smokers than in non-smokers.^72,369-372^ | Not significantly associated with LC risk.^104,373^  Independently associated with NSCLC^374^ and significantly higher in tumour tissues from NSCLC patients compared to normal lung tissues.^375^ | Significantly correlated with pack-years smoked.^72,369-372^ | Significantly reduced after 8 weeks’ cessation.^165^  Reduction observable after 1 weeks’ cessation.^376^ | N/A | 0/1 |
|  | Platelet count | One study has found platelet count is lower in smokers than non-smokers,^377^ but most studies find it is significantly higher in smokers than non-smokers.^378-383^ | There may be a casual relationship with LC risk.^384^  Higher than 326x10^9^/L in 40+yr olds significantly associated with increased odds of LC.^385^  Associated with poor prognosis in LC patients.^386,387^ | Strong dose-response relationship between platelet count and smoking intensity. ^131,383,388,389^ | One study found cessation increased platelet count.^377^  However, other studies found cessation reduces platelet count^131^, significantly so after 2 weeks,^390^ and may normalise to never smoker levels after around 2 years cessation.^131^ | 6 months | 4/5 |
|  | Soluble CD40 ligand | Significantly higher in smokers compared to non-smokers.^72,391^  Non-significantly higher in smokers than non-smokers. ^368,392,393^ | Higher levels in LC patients than healthy controls.^394,395^  Significantly correlated with metastatic spread of tumours in LC cells,^396^ and associated with advanced LC stages and poor prognosis.^395^ | Correlates with plasma cotinine levels.^72,100^  Not significantly associated with cigarettes-per-day or salivary cotinine levels in female smokers.^368^ | Not significantly different between smokers and smokers who quit for 6 weeks^368^ or 1 year.^81^ | IFE | 1/1 |
|  | Soluble P-selectin | Significantly higher in current smokers than non-smokers.^72-74,391^ | Significantly elevated in LC patients compared to healthy controls.^80^  Significantly associated with LC risk.^365^ | Strongly correlated with cigarettes-per-day, pack-years and plasma cotinine.^73,74^ | Significantly decreased after 6 weeks cessation.^100^  Lower in quitters and approached non-smoker levels following >20 years of cessation.^73^ | IFE | 1/1 |
| **Respiratory health and function** | Diffusion capacity | Significantly negatively associated with smoking.^397-403^ | Significantly lower in COPD patients with LC than those without.^404^  Not significantly different between idiopathic pulmonary fibrosis patients with and without LC.^405^ | Significantly negatively associated with smoking in a dose-dependent manner.^397-401^ | Improvement may be rapid following cessation,^403^with a significant increase observable after cessation for 1 week.^406^  However, one study observed no significant change after 1 year of cessation^407^and some suggest reduction may be irreversible and levels may not normalise following cessation. ^403,406^ | 3 months | 2/2 |
|  | Expiratory reserve volume | One study found it was significantly lower in smokers than non-smokers,^408^ but most observed no significant difference between smokers and non-smokers.^409-411^ | *No evidence on an association with LC found.* | *No evidence on a dose-response relationship found.* | *No evidence on reversibility found.* | IFE | 1/1 |
|  | Functional residual capacity | One study found no significant association with smoking,^399^ while others found there is a significant positive association.^398,412^ | Significantly higher in COPD patients with LC than those without. Static hyperinflation (FRC >120% pred value) is an independent risk factor for LC development.^413^ | Positively associated with pack-years.^398^ | *No evidence on reversibility found.* | 6 months | 2/3 |
|  | Oxygen saturation | Significantly lower in smokers than non-smokers.^397,414-418^ | Nocturnal oxygen desaturation significantly associated with LC incidence.^419-421^  No significant difference between LC patients and healthy controls.^422^ | Decreases with increased pack-years.^397^ | Amongst patients with COPD and nocturnal oxygen desaturation, percentage of SaO2 < 90% significantly decreased in quitters after 3 months’ cessation and was significantly lower than in active smokers.^423^  As exhaled carbon monoxide decreases, SaO2 increases.^424^ | IFE | 1/1 |
|  | Rate constant of CO/ CO transfer coefficient | Significantly lower in smokers than non-smokers.^400,402,425-427^ | *No evidence on an association with LC found.* | Significantly correlated with cotinine and cotinine x pack years, but not pack years.^426^ | Significantly increases following 6 weeks’ cessation.^428^  Significantly higher in ex-smokers than smokers^425,429^, and levels can approach those observed in never smokers after several years of abstinence. ^427,429^ | 3 months | 2/2 |
|  | Residual volume | Significantly positively associated with smoking.^399,430-432^ | Not significantly different between COPD patients with and without LC.^413^ | There is a dose-response association with pack-years.^398,432^ | Significantly reduced in quitters, ^432,433^ and may normalise to non-smoker levels within 5 years of cessation.^434^ | 6 months | 3/3 |
|  | Respiratory impedance | Significantly higher in smokers than non-smokers.^410,435-439^ | *No evidence on an association with LC found.* | Significantly correlated with pack-years.^436^ | No difference between never and ex-smokers.^440^ | IFE | 1/1 |
|  | Spirometry (FEV1, FVC, VC, FEV1/FVC, PEF, FEF[25-75%], IC) | Significantly worsened in smokers compared to non-smokers.^247,397,441-449^  Current smokers have higher likelihood of having obstructive spirometry pattern than never smokers.^450^ | Moderate or severe obstructive lung disease (defined as FEV1/FVC <70% & FEV1 <80% pred value) significantly associated with higher risk of LC incidence.^451^  A Mendelian randomisation study found no causality between FEV1 and LC risk,^452^ but most studies found reduced FEV1 strongly associated with increased LC risk.^453-455^  PEF is associated with LC mortality.^456-458^ | Smoking associated with worsening of all spirometry parameters in a dose-dependent manner.^397,399,442,444,445,448,459,460^ | Cessation significantly improves spirometry within months ^444-446,461,462^ and cessation duration is associated with reduced likelihood of obstructive spirometry.^450^  However, obstructive spirometry patterns still observed in ex-smokers even after several decades of cessation.^445,450^ | 6-12 months | 1-6/2-6 |
|  | Total lung capacity | Two studies found significant association with smoking,^398,463^ but others found no significant association with smoking.^399,428,430,464^ | Not significantly different between COPD patients with and without LC.^413^ | Significantly associated with pack-years.^398^ | Higher in ex-smokers than non-smokers, but lower than current smoker levels.^463^ | 6 months | 3/4 |
|  | Total respiratory resistance | Significantly increased following acute smoking.^410,465^  Significantly higher in smokers than non-smokers.^466^  No significant difference between smokers and non-smokers.^467-469^ | *No evidence on an association with LC found.* | *No evidence on a dose-response relationship found.* | Significantly higher in smokers than ex-smokers.^466^ | IFE | 1/1 |

# Supplementary Table 2. Summary of HTP clinical trials which report data on the most appropriate biomarkers for assessing lung cancer risk.

| **Trial ID** | **Design** | **Arms**  **(n participants completed)** | **Duration (setting)** | **Mode of Exposure** | **Biomarkers (contributing to syntheses)** |
| --- | --- | --- | --- | --- | --- |
| ISRCTN14301360/ UMIN000024988 | Randomised controlled - parallel | HTP, Glo (30)  HTP, menthol Glo (30)  HTP, IQOS (30)  CC, Lucky Strike Regular (30)  CC, Lucky Strike Menthol (30)  Cessation (30) | 5 days (confined) | Direct *ad libitum* | Monohydroxybutenyl mercapturic acid; 2-hydroxyethyl mercapturic acid; Nicotine equivalents; Total 4-(methyl nitrosamino)-1-(3-pyridyl)-1-butanol. |
| ISRCTN80651909 | Randomised controlled - parallel | HTP, Glo (28)  HTP, unknown brand (28)  CC, Lucky Strike Regular (30)  EC, IS1.0[TT] (30)  Cessation (29) | 5 days (confined) | Direct *ad libitum* | Monohydroxybutenyl mercapturic acid; 2-hydroxyethyl mercapturic acid; Nicotine equivalents; Total 4-(methyl nitrosamino)-1-(3-pyridyl)-1-butanol. |
| ISRCTN81075760 | Randomised controlled - parallel | HTP, Glo (127)  HTP, THD2.4T20 (0)  CC, own brand (59)  Cessation (109)  Never smokers (37) | 180 days  (ambulatory) | Direct *ad libitum* | Monohydroxybutenyl mercapturic acid; 2-hydroxyethyl mercapturic acid; Nicotine equivalents; Total 4-(methyl nitrosamino)-1-(3-pyridyl)-1-butanol; Nitric oxide; White blood cell count; 8-epi-prostaglandin F2alpha; Spirometry. |
| NCT01780714 | Randomised controlled - parallel | HTP, IQOS (20)  CC, own brand (20) | 5 days (confined) | Direct *ad libitum* | Monohydroxybutenyl mercapturic acid; Nicotine; Cotinine; Nicotine equivalents; Total 4-(methyl nitrosamino)-1-(3-pyridyl)-1-butanol. |
| NCT01959932 | Randomised controlled - parallel | HTP, IQOS (79)  CC, own brand (41)  Cessation (39) | 5 days (confined) | Direct *ad libitum* | Monohydroxybutenyl mercapturic acid; 2-hydroxyethyl mercapturic acid; Nicotine; Cotinine; Nicotine equivalents; Total 4-(methyl nitrosamino)-1-(3-pyridyl)-1-butanol. |
| NCT01970982 | Randomised controlled - parallel | HTP, IQOS (80)  CC, own brand (40)  Cessation (38) | 5 days (confined) | Direct *ad libitum* | Monohydroxybutenyl mercapturic acid; 2-hydroxyethyl mercapturic acid; Nicotine; Cotinine; Nicotine equivalents; Total 4-(methyl nitrosamino)-1-(3-pyridyl)-1-butanol. |
| NCT01970995 | Randomised controlled - parallel | HTP, IQOS menthol (76)  CC, own brand menthol (41)  Cessation (38) | 5 days (confined)  85 days (ambulatory) | Direct *ad libitum* | Monohydroxybutenyl mercapturic acid; 2-hydroxyethyl mercapturic acid; Nicotine; Cotinine; Nicotine equivalents; Total 4-(methyl nitrosamino)-1-(3-pyridyl)-1-butanol; White blood cell count; 8-epi-prostaglandin F2alpha. |
| NCT01989156 | Randomised controlled - parallel | HTP, IQOS menthol (73)  CC, own brand menthol (35)  Cessation (31) | 5 days (confined)  85 days (ambulatory) | Direct *ad libitum* | Monohydroxybutenyl mercapturic acid; 2-hydroxyethyl mercapturic acid; Nicotine; Cotinine; Nicotine equivalents; Total 4-(methyl nitrosamino)-1-(3-pyridyl)-1-butanol; White blood cell count; 8-epi-prostaglandin F2alpha. |
| NCT02396381 | Randomised controlled - parallel | HTP, IQOS (414)  CC, own brand (443) | 6 months (ambulatory) | Direct *ad libitum* | Monohydroxybutenyl mercapturic acid; Nicotine; Cotinine; Nicotine equivalents; Total 4-(methyl nitrosamino)-1-(3-pyridyl)-1-butanol; White blood cell count; Fibrinogen; Apolipoprotein; 8-epi-prostaglandin F2alpha; Spirometry. |
| NCT02503254 | Randomised controlled - parallel | HTP, CHTP1.0 (41)  CC, own brand (39) | 5 days (confined) | Direct *ad libitum* | Monohydroxybutenyl mercapturic acid; 2-hydroxyethyl mercapturic acid; Nicotine; Cotinine; Nicotine equivalents; Total 4-(methyl nitrosamino)-1-(3-pyridyl)-1-butanol. |
| NCT02641587 | Randomised controlled - parallel | HTP, CHTP1.2 (76)  CC, own brand (39) | 5 days (confined)  85 days (ambulatory) | Direct *ad libitum* | Monohydroxybutenyl mercapturic acid; 2-hydroxyethyl mercapturic acid; Nicotine equivalents; Total 4-(methyl nitrosamino)-1-(3-pyridyl)-1-butanol; White blood cell count; 8-epi-prostaglandin F2alpha. |
| NCT02649556 | Randomised controlled - parallel | HTP, IQOS (167)  CC, own brand (312) | 12 months | Direct *ad libitum* | Total 4-(methyl nitrosamino)-1-(3-pyridyl)-1-butanol; White blood cell count; 8-epi-prostaglandin F2alpha; Spirometry. |
| NCT03301129 | Randomised controlled - crossover | HTP, IQOS - CC, Marlboro Gold - EC, Blu Pro (20) | 3 single use sessions (confined) | Direct *ad libitum* | Vitamin E |
| NCT03364751 | Randomised controlled - parallel | HTP, IQOS (86)  CC, own brand (84) | 6 months (ambulatory) | Direct *ad libitum* | Nicotine equivalents; Total 4-(methyl nitrosamino)-1-(3-pyridyl)-1-butanol; Bleeding on Probing. |
| NCT03452124 | Randomised controlled - crossover + case-control study | RCT: HTP, IQOS - sham cigarette - CC, Marlboro Red (50)  Case-control: HTP, IQOS (50)  CC, unknown brand (25) | RCT: 3 7-min sessions  Case-control: 1 month | Direct *ad libitum* | Malondialdehyde |
| UMIN000025777 | Randomised controlled - parallel | HTP, NVTP (20)  CC, own brand (20)  Cessation (20) | 5 days (confined) | Direct *ad libitum* | Monohydroxybutenyl mercapturic acid; 4-hydroxybutyl-2-mercapturic acid; 2-hydroxyethyl mercapturic acid; Nicotine equivalents; Total 4-(methyl nitrosamino)-1-(3-pyridyl)-1-butanol. |
| UMIN000041539 | Randomised controlled - parallel | HTP, Ploom Tech+ (NR)  HTP, Ploom S2.0 (NR)  HTP, unknown brand (NR)  HTP, unknown brand (NR)  CC, own brand (NR)  Cessation (NR) | 5 days (confined) | Direct (unclear if *ad libitum*) | Monohydroxybutenyl mercapturic acid; 2-hydroxyethyl mercapturic acid; Total 4-(methyl nitrosamino)-1-(3-pyridyl)-1-butanol. |
| Abbreviations: HTP = heated tobacco product; CC = combustible cigarette; EC = electronic cigarette; own brand = participants’ own brand of cigarette; NR = not reported. | | | | | |

1. Frigerio G, Mercadante R, Campo L, et al. Urinary biomonitoring of subjects with different smoking habits. Part I: Profiling mercapturic acids. *Toxicology Letters.* 2020;327:48-57.

2. Hecht SS, Yuan J-M, Hatsukami D. Applying Tobacco Carcinogen and Toxicant Biomarkers in Product Regulation and Cancer Prevention. *Chemical Research in Toxicology.* 2010;23(6):1001-1008.

3. Roethig HJ, Munjal S, Feng S, et al. Population estimates for biomarkers of exposure to cigarette smoke in adult U.S. cigarette smokers. *Nicotine & Tobacco Research.* 2009;11(10):1216-1225.

4. Urban M, Gilch G, Schepers G, Miert Ev, Scherer G. Determination of the major mercapturic acids of 1,3-butadiene in human and rat urine using liquid chromatography with tandem mass spectrometry. *Journal of Chromatography B.* 2003;796(1):131-140.

5. Yuan J-M, Butler LM, Stepanov I, Hecht SS. Urinary Tobacco Smoke–Constituent Biomarkers for Assessing Risk of Lung Cancer. *Cancer Research.* 2014;74(2):401-411.

6. Yuan J-M, Gao Y-T, Wang R, Chen M, Carmella SG, Hecht SS. Urinary levels of volatile organic carcinogen and toxicant biomarkers in relation to lung cancer development in smokers. *Carcinogenesis.* 2012;33(4):804-809.

7. Carmella SG, Chen M, Han S, et al. Effects of Smoking Cessation on Eight Urinary Tobacco Carcinogen and Toxicant Biomarkers. *Chemical Research in Toxicology.* 2009;22(4):734-741.

8. Theophilus EH, Coggins CRE, Chen P, Schmidt E, Borgerding MF. Magnitudes of biomarker reductions in response to controlled reductions in cigarettes smoked per day: A one-week clinical confinement study. *Regulatory Toxicology and Pharmacology.* 2015;71(2):225-234.

9. Neophytou AM, Hart JE, Chang Y, et al. Short-Term Traffic-Related Exposures and Biomarkers of Nitro-PAH Exposure and Oxidative DNA Damage. *Toxics.* 2014;2(3):377-390.

10. Riedel K, Scherer G, Engl J, Hagedorn H-W, Tricker AR. Determination of Three Carcinogenic Aromatic Amines in Urine of Smokers and Nonsmokers. *Journal of Analytical Toxicology.* 2006;30(3):187-195.

11. Riffelmann M, Müller G, Schmieding W, Popp W, Norpoth K. Biomonitoring of urinary aromatic amines and arylamine hemoglobin adducts in exposed workers and nonexposed control persons. *Int Arch Occup Environ Health.* 1995;68(1):36-43.

12. Talhout R, Schulz T, Florek E, Van Benthem J, Wester P, Opperhuizen A. Hazardous Compounds in Tobacco Smoke. *Int J Environ Res Public Health.* 2011;8(2):613-628.

13. DeMarini DM. Genotoxicity of tobacco smoke and tobacco smoke condensate: a review. *Mutation Research/Reviews in Mutation Research.* 2004;567(2):447-474.

14. Hatsukami DK, Benowitz NL, Rennard SI, Oncken C, Hecht SS. Biomarkers to Assess the Utility of Potential Reduced Exposure Tobacco Products. *Nicotine & Tobacco Research.* 2006;8(2):169-191.

15. Airoldi L, Vineis P, Colombi A, et al. 4-Aminobiphenyl-Hemoglobin Adducts and Risk of Smoking-Related Disease in Never Smokers and Former Smokers in the European Prospective Investigation into Cancer and Nutrition Prospective Study. *Cancer Epidemiology, Biomarkers & Prevention.* 2005;14(9):2118-2124.

16. Vineis P, Caporaso N. Tobacco and cancer: epidemiology and the laboratory. *Environmental Health Perspectives.* 1995;103(2):156-160.

17. Weston A, Caporaso NE, Taghizadeh K, et al. Measurement of 4-Aminobiphenyl-Hemoglobin Adducts in Lung Cancer Cases and Controls. *Cancer Research.* 1991;51(19):5219-5223.

18. Alwis KU, Blount BC, Britt AS, Patel D, Ashley DL. Simultaneous analysis of 28 urinary VOC metabolites using ultra high performance liquid chromatography coupled with electrospray ionization tandem mass spectrometry (UPLC-ESI/MSMS). *Anal Chim Acta.* 2012;750:152-160.

19. Cigan S, Murphy S, Patel Y, et al. FP12.03 Associations of Urinary Biomarkers of Tobacco Toxicants With Lung Cancer Incidence in Smokers: The Multiethnic Cohort Study. *Journal of Thoracic Oncology.* 2021;16(10):S965.

20. Yuan J-M, Butler LM, Gao Y-T, et al. Urinary metabolites of a polycyclic aromatic hydrocarbon and volatile organic compounds in relation to lung cancer development in lifelong never smokers in the Shanghai Cohort Study. *Carcinogenesis.* 2013;35(2):339-345.

21. Habibagahi A, Alderman N, Kubwabo C. A review of the analysis of biomarkers of exposure to tobacco and vaping products. *Analytical Methods.* 2020;12(35):4276-4302.

22. Chung C-J, Hsu H-T, Chang C-H, et al. Relationships among cigarette smoking, urinary biomarkers, and urothelial carcinoma risk: a case-control study. *Environmental Science and Pollution Research.* 2020;27(34):43177-43185.

23. Hecht SS. Human urinary carcinogen metabolites: biomarkers for investigating tobacco and cancer. *Carcinogenesis.* 2002;23(6):907-922.

24. Hecht SS. Oral Cell DNA Adducts as Potential Biomarkers for Lung Cancer Susceptibility in Cigarette Smokers. *Chemical Research in Toxicology.* 2017;30(1):367-375.

25. Chang CM, Edwards SH, Arab A, Del Valle-Pinero AY, Yang L, Hatsukami DK. Biomarkers of Tobacco Exposure: Summary of an FDA-Sponsored Public Workshop. *Cancer Epidemiol Biomarkers Prev.* 2017;26(3):291-302.

26. Grigoryeva E, Kokova D, Gratchev A, et al. Smoking-related DNA adducts as potential diagnostic markers of lung cancer: new perspectives. *Experimental oncology.* 2015(37,№ 1):5-12.

27. Tombolini F, Pigini D, Tranfo G, et al. Levels of urinary metabolites of four PAHs and cotinine determined in 1016 volunteers living in Central Italy. *Environmental Science and Pollution Research.* 2018;25(29):28772-28779.

28. Park SL, Carmella SG, Chen M, et al. Mercapturic Acids Derived from the Toxicants Acrolein and Crotonaldehyde in the Urine of Cigarette Smokers from Five Ethnic Groups with Differing Risks for Lung Cancer. *PLOS ONE.* 2015;10(6):e0124841.

29. Sasson IM, Coleman DT, LaVoie EJ, Hoffmann D, Wynder EL. Mutagens in human urine: effects of cigarette smoking and diet. *Mutation Research/Genetic Toxicology.* 1985;158(3):149-157.

30. Lowe FJ, Luettich K, Gregg EO. Lung cancer biomarkers for the assessment of modified risk tobacco products: an oxidative stress perspective. *Biomarkers.* 2013;18(3):183-195.

31. Centers for Disease C, Prevention, National Center for Chronic Disease P, Health P, Office on S, Health. Publications and Reports of the Surgeon General. In: *How Tobacco Smoke Causes Disease: The Biology and Behavioral Basis for Smoking-Attributable Disease: A Report of the Surgeon General.* Atlanta (GA): Centers for Disease Control and Prevention (US); 2010.

32. Warren GW, Singh AK. Nicotine and lung cancer. *J Carcinog.* 2013;12:1.

33. Davis R, Rizwani W, Banerjee S, et al. Nicotine promotes tumor growth and metastasis in mouse models of lung cancer. *PLoS One.* 2009;4(10):e7524.

34. Joseph AM, Hecht SS, Murphy SE, et al. Relationships between cigarette consumption and biomarkers of tobacco toxin exposure. *Cancer Epidemiol Biomarkers Prev.* 2005;14(12):2963-2968.

35. Rostron BL, Wang J, Etemadi A, et al. Associations between Biomarkers of Exposure and Lung Cancer Risk among Exclusive Cigarette Smokers in the Golestan Cohort Study. *Int J Environ Res Public Health.* 2021;18(14):7349.

36. Kawanishi S, Hiraku Y, Murata M, Oikawa S. The role of metals in site-specific DNA damage with reference to carcinogenesis1, 2 1Guest Editor: Miral Dizdaroglu 2This article is part of a series of reviews on “Oxidative DNA Damage and Repair.” The full list of papers may be found on the homepage of the journal. *Free Radical Biology and Medicine.* 2002;32(9):822-832.

37. Park SL, Murphy SE, Wilkens LR, Stram DO, Hecht SS, Le Marchand L. Association of CYP2A6 activity with lung cancer incidence in smokers: The multiethnic cohort study. *PLOS ONE.* 2017;12(5):e0178435.

38. Mwenifumbo JC, Sellers EM, Tyndale RF. Nicotine metabolism and CYP2A6 activity in a population of black African descent: Impact of gender and light smoking. *Drug and Alcohol Dependence.* 2007;89(1):24-33.

39. Sinues B, Fanlo A, Mayayo E, et al. CYP2A6 activity in a healthy Spanish population: effect of age, sex, smoking, and oral contraceptives. *Human & Experimental Toxicology.* 2008;27(5):367-372.

40. Tanner J-A, Tyndale RF. Variation in CYP2A6 Activity and Personalized Medicine. *Journal of Personalized Medicine.* 2017;7(4):18.

41. Benowitz NL, Pomerleau OF, Pomerleau CS, Jacob P, III. Nicotine metabolite ratio as a predictor of cigarette consumption. *Nicotine & Tobacco Research.* 2003;5(5):621-624.

42. EOM SY, YIM DH, MOON SI, et al. Polycyclic Aromatic Hydrocarbon-induced Oxidative Stress, Antioxidant Capacity, and the Risk of Lung Cancer: A Pilot Nested Case-control Study. *Anticancer Research.* 2013;33(8):3089-3097.

43. Zhang H, Lu H, Huang H, et al. Quantification of 1-hydroxypyrene in undiluted human urine samples using magnetic solid-phase extraction coupled with internal extractive electrospray ionization mass spectrometry. *Analytica Chimica Acta.* 2016;926:72-78.

44. Van Rooij JG, Veeger MM, Bodelier-Bade MM, Scheepers PT, Jongeneelen FJ. Smoking and dietary intake of polycyclic aromatic hydrocarbons as sources of interindividual variability in the baseline excretion of 1-hydroxypyrene in urine. *Int Arch Occup Environ Health.* 1994;66(1):55-65.

45. Minet E, Cheung F, Errington G, Sterz K, Scherer G. Urinary excretion of the acrylonitrile metabolite 2-cyanoethylmercapturic acid is correlated with a variety of biomarkers of tobacco smoke exposure and consumption. *Biomarkers.* 2011;16(1):89-96.

46. Shie HG, Pan SW, Yu WK, Chen WC, Ho LI, Ko HK. Levels of exhaled carbon monoxide measured during an intervention program predict 1-year smoking cessation: a retrospective observational cohort study. *NPJ Prim Care Respir Med.* 2017;27(1):59.

47. Siemann DW, Hill RP, Bush RS. Smoking: The influence of carboxyhemoglobin (HbCO) on tumor oxygenation and response to radiation. *International Journal of Radiation Oncology*Biology*Physics.* 1978;4(7):657-662.

48. Yasuda H, Yamaya M, Ebihara S, et al. ARTERIAL CARBOXYHEMOGLOBIN CONCENTRATIONS IN ELDERLY PATIENTS WITH OPERABLE NON-SMALL CELL LUNG CANCER. *Journal of the American Geriatrics Society.* 2004;52(9):1592-1593.

49. Kambam JR, Chen LH, Hyman SA. Effect of short-term smoking halt on carboxyhemoglobin levels and P50 values. *Anesth Analg.* 1986;65(11):1186-1188.

50. Fennell TR, MacNeela JP, Morris RW, Watson M, Thompson CL, Bell DA. Hemoglobin Adducts from Acrylonitrile and Ethylene Oxide in Cigarette Smokers: Effects of Glutathione S-Transferase T1-Null and M1-Null Genotypes. *Cancer Epidemiology, Biomarkers & Prevention.* 2000;9(7):705-712.

51. Schettgen T, Rossbach B, Kütting B, Letzel S, Drexler H, Angerer J. Determination of haemoglobin adducts of acrylamide and glycidamide in smoking and non-smoking persons of the general population. *International Journal of Hygiene and Environmental Health.* 2004;207(6):531-539.

52. Schettgen T, Musiol A, Alt A, Kraus T. Fast determination of urinary S-phenylmercapturic acid (S-PMA) and S-benzylmercapturic acid (S-BMA) by column-switching liquid chromatography–tandem mass spectrometry. *Journal of Chromatography B.* 2008;863(2):283-292.

53. Qin N, Zhu Y, Zhong Y, et al. External Exposure to BTEX, Internal Biomarker Response, and Health Risk Assessment of Nonoccupational Populations near a Coking Plant in Southwest China. *Int J Environ Res Public Health.* 2022;19(2):847.

54. Bakhru A, Erlinger TP. Smoking Cessation and Cardiovascular Disease Risk Factors: Results from the Third National Health and Nutrition Examination Survey. *PLOS Medicine.* 2005;2(6):e160.

55. Kühn T, Sookthai D, Graf ME, et al. Albumin, bilirubin, uric acid and cancer risk: results from a prospective population-based study. *Br J Cancer.* 2017;117(10):1572-1579.

56. Sprague BL, Trentham-Dietz A, Klein BEK, et al. Physical Activity, White Blood Cell Count, and Lung Cancer Risk in a Prospective Cohort Study. *Cancer Epidemiology, Biomarkers & Prevention.* 2008;17(10):2714-2722.

57. Yang Z, Zheng Y, Wu Z, et al. Association between pre-diagnostic serum albumin and cancer risk: Results from a prospective population-based study. *Cancer Medicine.* 2021;10(12):4054-4065.

58. Yoon HS, Shu XO, Shidal C, et al. Associations of Pre-Diagnostic Serum Levels of Total Bilirubin and Albumin With Lung Cancer Risk: Results From the Southern Community Cohort Study. *Front Oncol.* 2022;12:895479.

59. Shaper AG, Wannamethee SG, Whincup PH. Serum albumin and risk of stroke, coronary heart disease, and mortality: the role of cigarette smoking. *Journal of Clinical Epidemiology.* 2004;57(2):195-202.

60. Wannamethee SG, Lowe GDO, Shaper AG, Rumley A, Lennon L, Whincup PH. Associations between cigarette smoking, pipe/cigar smoking, and smoking cessation, and haemostatic and inflammatory markers for cardiovascular disease. *European Heart Journal.* 2005;26(17):1765-1773.

61. Sköld CM, Blaschke E, Eklund A. Transient increases in albumin and hyaluronan in bronchoalveolar lavage fluid after quitting smoking: possible signs of reparative mechanisms. *Respiratory Medicine.* 1996;90(9):523-529.

62. Klarlund M, Priemé H, Loft S, Poulsen HE. Smoking cessation does not change urinary albumin excretion in normal subjects. *Scandinavian Journal of Clinical and Laboratory Investigation.* 1997;57(6):513-520.

63. Haak T, Jungmann E, Raab C, Usadel KH. Elevated endothelin-1 levels after cigarette smoking. *Metabolism.* 1994;43(3):267-269.

64. Hirai Y, Adachi H, Fujiura Y, Hiratsuka A, Enomoto M, Imaizumi T. Plasma endothelin-1 level is related to renal function and smoking status but not to blood pressure: an epidemiological study. *J Hypertens.* 2004;22(4):713-718.

65. Goerre S, Staehli C, Shaw S, Lüscher TF. Effect of cigarette smoking and nicotine on plasma endothelin-1 levels. *J Cardiovasc Pharmacol.* 1995;26 Suppl 3:S236-238.

66. Zhang W-M, Zhou J, Ye Q-J. Endothelin-1 enhances proliferation of lung cancer cells by increasing intracellular free Ca2+. *Life Sciences.* 2008;82(13):764-771.

67. Cohen A. Endothelin receptor expression in lung cancer cell lines and bronchial epithelial cell lines. *The FASEB journal.* 1997;11(3):3221.

68. Bagnato A, Spinella F. Emerging role of endothelin-1 in tumor angiogenesis. *Trends in Endocrinology & Metabolism.* 2003;14(1):44-50.

69. Chen JL, Lv XD, Ma H, Chen JR, Huang JA. Detection of cancer embryo antigen and endothelin-1 in exhaled breath condensate: A novel approach to investigate non-small cell lung cancer. *Mol Clin Oncol.* 2016;5(1):124-128.

70. Carpagnano GE, Foschino-Barbaro MP, Resta O, Gramiccioni E, Carpagnano F. Endothelin-1 Is Increased in the Breath Condensate of Patients with Non-Small-Cell Lung Cancer. *Oncology.* 2004;66(3):180-184.

71. Derella CC, Tingen MS, Blanks A, et al. Smoking cessation reduces systemic inflammation and circulating endothelin-1. *Scientific Reports.* 2021;11(1):24122.

72. Yanbaeva DG, Dentener MA, Creutzberg EC, Wesseling G, Wouters EF. Systemic effects of smoking. *Chest.* 2007;131(5):1557-1566.

73. Delgado GE, Krämer BK, Siekmeier R, et al. Influence of smoking and smoking cessation on biomarkers of endothelial function and their association with mortality. *Atherosclerosis.* 2020;292:52-59.

74. Demerath E, Towne B, Blangero J, Siervogel RM. The relationship of soluble ICAM-1, VCAM-1, P-selectin and E-selectin to cardiovascular disease risk factors in healthy men and women. *Annals of Human Biology.* 2001;28(6):664-678.

75. Kumboyono K, Nurwidyaningtyas W, Chomsy IN, Wihastuti TA. Early Detection of Negative Smoking Impacts: Vascular Adaptation Deviation Based on Quantification of Circulated Endothelial Activation Markers. *Vasc Health Risk Manag.* 2021;17:103-109.

76. Hwang SJ, Ballantyne CM, Sharrett AR, et al. Circulating adhesion molecules VCAM-1, ICAM-1, and E-selectin in carotid atherosclerosis and incident coronary heart disease cases: the Atherosclerosis Risk In Communities (ARIC) study. *Circulation.* 1997;96(12):4219-4225.

77. Ponthieux A, Herbeth B, Droesch S, Haddy N, Lambert D, Visvikis S. Biological determinants of serum ICAM-1, E-selectin, P-selectin and L-selectin levels in healthy subjects: the Stanislas study. *Atherosclerosis.* 2004;172(2):299-308.

78. Gogali A, Charalabopoulos K, Zampira I, et al. Soluble Adhesion Molecules E-Cadherin, Intercellular Adhesion Molecule-1, and E-Selectin as Lung Cancer Biomarkers. *Chest.* 2010;138(5):1173-1179.

79. Swellam M, Ragab HM, Abdalla NA, El-Asmar A-BH. Soluble cytokeratin-19 and E-selectin biomarkers: Their relevance for lung cancer detection when tested independently or in combinations. *Cancer Biomarkers.* 2008;4:43-54.

80. Roselli M, Mineo TC, Martini F, et al. Soluble Selectin Levels in Patients with Lung Cancer. *The International Journal of Biological Markers.* 2002;17(1):56-62.

81. Halvorsen B, Sagen LE, Ueland T, Aukrust P, Tonstad S. Effect of smoking cessation on markers of inflammation and endothelial cell activation among individuals with high risk for cardiovascular disease. *Scandinavian Journal of Clinical and Laboratory Investigation.* 2007;67(6):604-611.

82. Messner B, Bernhard D. Smoking and Cardiovascular Disease. *Arteriosclerosis, Thrombosis, and Vascular Biology.* 2014;34(3):509-515.

83. Celermajer DS, Sorensen KE, Georgakopoulos D, et al. Cigarette smoking is associated with dose-related and potentially reversible impairment of endothelium-dependent dilation in healthy young adults. *Circulation.* 1993;88(5):2149-2155.

84. Johnson Heather M, Gossett Linda K, Piper Megan E, et al. Effects of Smoking and Smoking Cessation on Endothelial Function. *Journal of the American College of Cardiology.* 2010;55(18):1988-1995.

85. Robbins RA, Millatmal T, Lassi K, Rennard S, Daughton D. Smoking cessation is associated with an increase in exhaled nitric oxide. *Chest.* 1997;112(2):313-318.

86. Kharitonov SA, Robbins RA, Yates D, Keatings V, Barnes PJ. Acute and chronic effects of cigarette smoking on exhaled nitric oxide. *Am J Respir Crit Care Med.* 1995;152(2):609-612.

87. HÖGMAN M, HOLMKVIST T, WÅLINDER R, et al. Increased nitric oxide elimination from the airways after smoking cessation. *Clinical Science.* 2002;103(1):15-19.

88. Herath P, Wimalasekera S, Amarasekara T, Fernando M, Turale S. Effect of cigarette smoking on smoking biomarkers, blood pressure and blood lipid levels among Sri Lankan male smokers. *Postgraduate Medical Journal.* 2022;98(1165):848-854.

89. Grassi D, Desideri G, Ferri L, Aggio A, Tiberti S, Ferri C. Oxidative Stress and Endothelial Dysfunction: Say NO to Cigarette Smoking! *Current Pharmaceutical Design.* 2010;16(23):2539-2550.

90. Toda N, Toda H. Nitric oxide-mediated blood flow regulation as affected by smoking and nicotine. *European Journal of Pharmacology.* 2010;649(1):1-13.

91. Carnevale R, Sciarretta S, Violi F, et al. Acute Impact of Tobacco vs Electronic Cigarette Smoking on Oxidative Stress and Vascular Function. *Chest.* 2016;150(3):606-612.

92. Balint B, Donnelly LE, Hanazawa T, Kharitonov SA, Barnes PJ. Increased nitric oxide metabolites in exhaled breath condensate after exposure to tobacco smoke. *Thorax.* 2001;56(6):456-461.

93. Ghasemi A, Zahedi Asl S, Mehrabi Y, Saadat N, Azizi F. Serum nitric oxide metabolite levels in a general healthy population: Relation to sex and age. *Life Sciences.* 2008;83(9):326-331.

94. Zhou JF, Yan XF, Guo FZ, Sun NY, Qian ZJ, Ding DY. Effects of cigarette smoking and smoking cessation on plasma constituents and enzyme activities related to oxidative stress. *Biomed Environ Sci.* 2000;13(1):44-55.

95. Masri FA, Comhair SAA, Koeck T, et al. Abnormalities in Nitric Oxide and Its Derivatives in Lung Cancer. *American Journal of Respiratory and Critical Care Medicine.* 2005;172(5):597-605.

96. Zhou H, Li J, Chen Z, Chen Y, Ye S. Nitric oxide in occurrence, progress and therapy of lung Cancer: a systemic review and meta-analysis. *BMC Cancer.* 2021;21(1):678.

97. Liu CY, Wang CH, Chen TC, Lin HC, Yu CT, Kuo HP. Increased level of exhaled nitric oxide and up-regulation of inducible nitric oxide synthase in patients with primary lung cancer. *Br J Cancer.* 1998;78(4):534-541.

98. Masri F. Role of nitric oxide and its metabolites as potential markers in lung cancer. *Ann Thorac Med.* 2010;5(3):123-127.

99. Scott DA, Stapleton JA, Wilson RF, et al. Dramatic Decline in Circulating Intercellular Adhesion Molecule-1 Concentration on Quitting Tobacco Smoking. *Blood Cells, Molecules, and Diseases.* 2000;26(3):255-258.

100. Blann AD, Steele C, McCollum CN. THE INFLUENCE OF SMOKING ON SOLUBLE ADHESION MOLECULES AND ENDOTHELIAL CELL MARKERS. *Thrombosis Research.* 1997;85(5):433-438.

101. Lüdicke F, Magnette J, Baker G, Weitkunat R. A Japanese cross-sectional multicentre study of biomarkers associated with cardiovascular disease in smokers and non-smokers. *Biomarkers.* 2015;20(6-7):411-421.

102. Gu X, Ma C, Yuan D, Song Y. Circulating soluble intercellular adhesion molecule-1 in lung cancer: a systematic review. *Transl Lung Cancer Res.* 2012;1(1):36-44.

103. Osaki T, Mitsudomi T, Yoshida Y, et al. Increased levels of serum intercellular adhesion molecule-1 (ICAM-1) in patients with non-small cell lung cancer. *Surgical Oncology.* 1996;5(3):107-113.

104. Bouras E, Karhunen V, Gill D, et al. Circulating inflammatory cytokines and risk of five cancers: a Mendelian randomization analysis. *BMC Medicine.* 2022;20(1):3.

105. Palmer RM, Stapleton JA, Sutherland G, Coward PY, Wilson RF, Scott DA. Effect of nicotine replacement and quitting smoking on circulating adhesion molecule profiles (sICAM-1, sCD44v5, sCD44v6). *European Journal of Clinical Investigation.* 2002;32(11):852-857.

106. Tsai J-S, Guo F-R, Chen S-C, et al. Changes of serum adiponectin and soluble intercellular adhesion molecule-1 concentrations after smoking cessation. *Clinical Chemistry and Laboratory Medicine.* 2012;50(6):1063-1069.

107. Xue C, Chen QZ, Bian L, et al. Effects of Smoking Cessation with Nicotine Replacement Therapy on Vascular Endothelial Function, Arterial Stiffness, and Inflammation Response in Healthy Smokers. *Angiology.* 2019;70(8):719-725.

108. Kuschner W, D'Alessandro A, Wong H, Blanc P. Dose-dependent cigarette smoking-related inflammatory responses in healthy adults. *European Respiratory Journal.* 1996;9(10):1989-1994.

109. Tan Z, Xue H, Sun Y, Zhang C, Song Y, Qi Y. The Role of Tumor Inflammatory Microenvironment in Lung Cancer. *Frontiers in Pharmacology.* 2021;12.

110. Gschwandtner M, Derler R, Midwood KS. More Than Just Attractive: How CCL2 Influences Myeloid Cell Behavior Beyond Chemotaxis. *Frontiers in Immunology.* 2019;10.

111. Shiels MS, Shu X-O, Chaturvedi AK, et al. A prospective study of immune and inflammation markers and risk of lung cancer among female never smokers in Shanghai. *Carcinogenesis.* 2017;38(10):1004-1010.

112. Li X, Lin F, Zhou H. Genetic polymorphism rs3760396 of the chemokine (C-C motif) ligand 2 gene (CCL2) associated with the susceptibility of lung cancer in a pathological subtype-specific manner in Han-ancestry Chinese: a case control study. *BMC Cancer.* 2016;16(1):298.

113. Bazzano L, He J, Muntner P, Vupputuri S, Whelton P. Relationship between Cigarette Smoking and Novel Risk Factors for Cardiovascular Disease in the United States. *Annals of Internal Medicine.* 2003;138(11):891-897.

114. Shiels MS, Pfeiffer RM, Hildesheim A, et al. Circulating Inflammation Markers and Prospective Risk for Lung Cancer. *JNCI: Journal of the National Cancer Institute.* 2013;105(24):1871-1880.

115. Allin KH, Bojesen SE, Nordestgaard BG. Baseline C-Reactive Protein Is Associated With Incident Cancer and Survival in Patients With Cancer. *Journal of Clinical Oncology.* 2009;27(13):2217-2224.

116. Il'yasova D, Colbert LH, Harris TB, et al. Circulating Levels of Inflammatory Markers and Cancer Risk in the Health Aging and Body Composition Cohort. *Cancer Epidemiology, Biomarkers & Prevention.* 2005;14(10):2413-2418.

117. Shiels MS, Katki HA, Hildesheim A, et al. Circulating Inflammation Markers, Risk of Lung Cancer, and Utility for Risk Stratification. *JNCI: Journal of the National Cancer Institute.* 2015;107(10).

118. Zhou B, Liu J, Wang Z-M, Xi T. C-Reactive Protein, Interleukin 6 and Lung Cancer Risk: A Meta-Analysis. *PLOS ONE.* 2012;7(8):e43075.

119. Chaturvedi AK, Caporaso NE, Katki HA, et al. C-Reactive Protein and Risk of Lung Cancer. *Journal of Clinical Oncology.* 2010;28(16):2719-2726.

120. Gallus S, Lugo A, Suatoni P, et al. Effect of Tobacco Smoking Cessation on C-Reactive Protein Levels in A Cohort of Low-Dose Computed Tomography Screening Participants. *Sci Rep.* 2018;8(1):12908.

121. Hastie CE, Haw S, Pell JP. Impact of Smoking Cessation and Lifetime Exposure on C-Reactive Protein. *Nicotine & Tobacco Research.* 2008;10(4):637-642.

122. Zahed H, Johansson M, Ueland PM, et al. Epidemiology of 40 blood biomarkers of one-carbon metabolism, vitamin status, inflammation, and renal and endothelial function among cancer-free older adults. *Scientific Reports.* 2021;11(1):13805.

123. Nygård O, Vollset SE, Refsum H, et al. Total Plasma Homocysteine and Cardiovascular Risk Profile: The Hordaland Homocysteine Study. *JAMA.* 1995;274(19):1526-1533.

124. Sobczak A, Wardas W, Zielinska-Danch W, Pawlicki K. The influence of smoking on plasma homocysteine and cysteine levels in passive and active smokers. *Clinical Chemistry and Laboratory Medicine (CCLM).* 2004;42(4):408-414.

125. Wu LL, Wu JT. Hyperhomocysteinemia is a risk factor for cancer and a new potential tumor marker. *Clinica Chimica Acta.* 2002;322(1):21-28.

126. Stanisławska-Sachadyn A, Borzyszkowska J, Krzemiński M, et al. Folate/homocysteine metabolism and lung cancer risk among smokers. *PLOS ONE.* 2019;14(4):e0214462.

127. Tonstad S, Urdal P. Does short-term smoking cessation reduce plasma total homocysteine concentrations? *Scandinavian Journal of Clinical and Laboratory Investigation.* 2002;62(4):279-284.

128. Pedersen KM, Çolak Y, Ellervik C, Hasselbalch HC, Bojesen SE, Nordestgaard BG. Smoking and Increased White and Red Blood Cells. *Arteriosclerosis, Thrombosis, and Vascular Biology.* 2019;39(5):965-977.

129. Phan T, Nguyen A, Nguyen A, et al. Neutrophil to lymphocyte with monocyte to lymphocyte ratio and white blood cell count in prediction of lung cancer. *Australasian Medical Journal.* 2018;11.

130. Wong JYY, Bassig BA, Loftfield E, et al. White Blood Cell Count and Risk of Incident Lung Cancer in the UK Biobank. *JNCI Cancer Spectrum.* 2019;4(2).

131. Van Tiel ED, Peeters PHM, Smit HA, et al. Quitting Smoking May Restore Hematological Characteristics within Five Years. *Annals of Epidemiology.* 2002;12(6):378-388.

132. Higuchi T, Omata F, Tsuchihashi K, Higashioka K, Koyamada R, Okada S. Current cigarette smoking is a reversible cause of elevated white blood cell count: Cross-sectional and longitudinal studies. *Prev Med Rep.* 2016;4:417-422.

133. Smith CJ, Kluck LA, Ruan GJ, et al. Leukocytosis and Tobacco Use: An Observational Study of Asymptomatic Leukocytosis. *The American Journal of Medicine.* 2021;134(1):e31-e35.

134. Kauss AR, Antunes M, de La Bourdonnaye G, et al. Smoking and apolipoprotein levels: A meta-analysis of published data. *Toxicology Reports.* 2022;9:1150-1171.

135. Craig WY, Palomaki GE, Haddow JE. Cigarette smoking and serum lipid and lipoprotein concentrations: an analysis of published data. *British Medical Journal.* 1989;298(6676):784-788.

136. Berg K, Börresen A-L, Dahlen G. Effect of smoking on serum levels of HDL apoproteins. *Atherosclerosis.* 1979;34(3):339-343.

137. Mazidi M, Katsiki N, Mikhailidis DP, Radenkovic D, Pella D, Banach M. Apolipoprotein B/Apolipoprotein A-I Ratio Is a Better Predictor of Cancer Mortality Compared with C-Reactive Protein: Results from Two Multi-Ethnic US Populations. *Journal of Clinical Medicine.* 2020;9(1):170.

138. Dong Y, Wang H, Shan D, Yu Z. [Research Progress on the Relationship between Blood Lipids and  Lung Cancer Risk and Prognosis]. *Zhongguo Fei Ai Za Zhi.* 2020;23(9):824-829.

139. Omvik P. How smoking affects blood pressure. *Blood Pressure.* 1996;5(2):71-77.

140. Berglund G, Wilhelmsen L. Factors Related to Blood Pressure in a General Population Sample of Swedish Men. *Acta Medica Scandinavica.* 1975;198(1-6):291-298.

141. Seltzer CC. Effect of smoking on blood pressure. *American Heart Journal.* 1974;87(5):558-564.

142. Green MS, Jucha E, Luz Y. Blood pressure in smokers and nonsmokers: Epidemiologic findings. *American Heart Journal.* 1986;111(5):932-940.

143. Green MS, Harari G. A prospective study of the effects of changes in smoking habits on blood count, serum lipids and lipoproteins, body weight and blood pressure in occupationally active men. The Israeli CORDIS study. *Journal of Clinical Epidemiology.* 1995;48(9):1159-1166.

144. Mandraffino G, Aragona CO, Scuruchi M, et al. Biglycan expression, earlier vascular damage and pro-atherogenic profile improvement after smoke cessation in young people. *Atherosclerosis.* 2017;257:109-115.

145. Primatesta P, Falaschetti E, Gupta S, Marmot MG, Poulter NR. Association Between Smoking and Blood Pressure. *Hypertension.* 2001;37(2):187-193.

146. Greene SB, Aavedal MJ, Tyroler HA, Davis CE, Hames CG. Smoking habits and blood pressure change: A seven year follow-up. *Journal of Chronic Diseases.* 1977;30(7):401-413.

147. Stocks T, Hemelrijck MV, Manjer J, et al. Blood Pressure and Risk of Cancer Incidence and Mortality in the Metabolic Syndrome and Cancer Project. *Hypertension.* 2012;59(4):802-810.

148. Cho IY, Han K, Shin DW, Cho MH, Yoo JE, Cho JH. Associations of Variability in Metabolic Parameters with Lung Cancer: A Nationwide Population-Based Study. *Cancers.* 2021;13(8):1982.

149. Grove JS, Nomura A, Severson RK, Stemmermann GN. The Association of Blood Pressure with Cancer Incidence in a Prospective Study. *American Journal of Epidemiology.* 1991;134(9):942-947.

150. Ding J, Tu Z, Chen H, Liu Z. Identifying modifiable risk factors of lung cancer: Indications from Mendelian randomization. *PLOS ONE.* 2021;16(10):e0258498.

151. Andriani H, Kosasih RI, Putri S, Kuo H-W. Effects of changes in smoking status on blood pressure among adult males and females in Indonesia: a 15-year population-based cohort study. *BMJ Open.* 2020;10(4):e038021.

152. Friedman GD, Siegelaub AB. Changes after quitting cigarette smoking. *Circulation.* 1980;61(4):716-723.

153. Puddey IB, Vandongen R, Beilino LJ, English DR, Ukich AW. The effect of stopping smoking on blood pressure—A controlled trial. *Journal of Chronic Diseases.* 1985;38(6):483-493.

154. Bornemisza P, Suciu I. Effect of cigarette smoking on the blood glucose level in normals and diabetics. *Med Interne.* 1980;18(4):353-356.

155. Śliwińska-Mossoń M, Milnerowicz H. The impact of smoking on the development of diabetes and its complications. *Diabetes and Vascular Disease Research.* 2017;14(4):265-276.

156. Sandberg H, Roman L, Zavodnick J, Kupers N. The effect of smoking on serum somatotropin, immunoreactive insulin and blood glucose levels of young adult males. *J Pharmacol Exp Ther.* 1973;184(3):787-791.

157. Cichosz SL, Jensen MH, Hejlesen O. Associations between smoking, glucose metabolism and lipid levels: A cross-sectional study. *Journal of Diabetes and its Complications.* 2020;34(10):107649.

158. Colberg SR, Casazza GA, Horning MA, Brooks GA. Increased dependence on blood glucose in smokers during rest and sustained exercise. *Journal of Applied Physiology.* 1994;76(1):26-32.

159. Harris KK, Zopey M, Friedman TC. Metabolic effects of smoking cessation. *Nature Reviews Endocrinology.* 2016;12(5):299-308.

160. Argirion I, Weinstein SJ, Männistö S, Albanes D, Mondul AM. Serum Insulin, Glucose, Indices of Insulin Resistance, and Risk of Lung Cancer. *Cancer Epidemiol Biomarkers Prev.* 2017;26(10):1519-1524.

161. Wang L, Si S, Li J, et al. Triglyceride-Glucose Index Is Not Associated With Lung Cancer Risk: A Prospective Cohort Study in the UK Biobank. *Front Oncol.* 2021;11:774937.

162. Yan X, Gao Y, Tong J, Tian M, Dai J, Zhuang Y. Association Between Triglyceride Glucose Index and Non-Small Cell Lung Cancer Risk in Chinese Population. *Frontiers in Oncology.* 2021;11.

163. Nakanishi N, Nakamura K, Matsuo Y, Suzuki K, Tatara K. Cigarette Smoking and Risk for Impaired Fasting Glucose and Type 2 Diabetes in Middle-Aged Japanese Men. *Annals of Internal Medicine.* 2000;133(3):183-191.

164. Tamura U, Tanaka T, Okamura T, et al. Changes in Weight, cardiovascular risk factors and estimated risk of coronary heart disease following smoking cessation in Japanese male workers: HIPOP-OHP study. *J Atheroscler Thromb.* 2010;17(1):12-20.

165. ELIASSON B, ATTVALL S, TASKINEN M-R, SMITH U. Smoking cessation improves insulin sensitivity in healthy middle-aged men. *European Journal of Clinical Investigation.* 1997;27(5):450-456.

166. Choi D-W, Jeon J, Lee SA, Han K-T, Park E-C, Jang S-I. Association between Smoking Behavior Patterns and Glycated Hemoglobin Levels in a General Population. *Int J Environ Res Public Health.* 2018;15(10):2260.

167. Kar D, Gillies C, Zaccardi F, et al. Relationship of cardiometabolic parameters in non-smokers, current smokers, and quitters in diabetes: a systematic review and meta-analysis. *Cardiovascular Diabetology.* 2016;15(1):158.

168. Srour B, Kaaks R, Johnson T, Hynes LC, Kühn T, Katzke VA. Ageing-related markers and risks of cancer and cardiovascular disease: a prospective study in the EPIC-Heidelberg cohort. *European Journal of Epidemiology.* 2022;37(1):49-65.

169. Mathur A, Nag OP. Serum HbA1C–a Marker for Lung Cancer? 2020.

170. de Beer JC, Liebenberg L. Does cancer risk increase with HbA1c, independent of diabetes? *Br J Cancer.* 2014;110(9):2361-2368.

171. Travier N, Jeffreys M, Brewer N, et al. Association between glycosylated hemoglobin and cancer risk: a New Zealand linkage study. *Annals of Oncology.* 2007;18(8):1414-1419.

172. Peila R, Rohan TE. Diabetes, Glycated Hemoglobin, and Risk of Cancer in the UK Biobank Study. *Cancer Epidemiology, Biomarkers & Prevention.* 2020;29(6):1107-1119.

173. Jain RB, Ducatman A. Associations between smoking and lipid/lipoprotein concentrations among US adults aged ≥20 years. *Journal of Circulating Biomarkers.* 2018;7:1849454418779310.

174. Willett W, Hennekens CH, Castelli W, et al. Effects of cigarette smoking on fasting triglyceride, total cholesterol, and HDL-cholesterol in women. *American Heart Journal.* 1983;105(3):417-421.

175. Zabłocka-Słowińska K, Płaczkowska S, Skórska K, et al. Oxidative stress in lung cancer patients is associated with altered serum markers of lipid metabolism. *PLOS ONE.* 2019;14(4):e0215246.

176. Hao B, Yu M, Sang C, Bi B, Chen J. Dyslipidemia and non-small cell lung cancer risk in Chinese population: a case-control study. *Lipids in Health and Disease.* 2018;17(1):278.

177. Ahn J, Lim U, Weinstein SJ, et al. Prediagnostic total and high-density lipoprotein cholesterol and risk of cancer. *Cancer Epidemiol Biomarkers Prev.* 2009;18(11):2814-2821.

178. Borgquist S, Butt T, Almgren P, et al. Apolipoproteins, lipids and risk of cancer. *International Journal of Cancer.* 2016;138(11):2648-2656.

179. Lin X, Lu L, Liu L, et al. Blood lipids profile and lung cancer risk in a meta-analysis of prospective cohort studies. *Journal of Clinical Lipidology.* 2017;11(4):1073-1081.

180. Lyu Z, Li N, Wang G, et al. Independent and joint associations of blood lipids and lipoproteins with lung cancer risk in Chinese males: A prospective cohort study. *International Journal of Cancer.* 2019;144(12):2972-2984.

181. Garrison RJ, Kannel WB, Feinleib M, Castelli WP, McNamara PM, Padgett SJ. Cigarette smoking and HDL cholesterol the Framingham offspring study. *Atherosclerosis.* 1978;30(1):17-25.

182. Maeda K, Noguchi Y, Fukui T. The effects of cessation from cigarette smoking on the lipid and lipoprotein profiles: a meta-analysis. *Preventive Medicine.* 2003;37(4):283-290.

183. Gepner AD, Piper ME, Johnson HM, Fiore MC, Baker TB, Stein JH. Effects of smoking and smoking cessation on lipids and lipoproteins: Outcomes from a randomized clinical trial. *American Heart Journal.* 2011;161(1):145-151.

184. Forey BA, Fry JS, Lee PN, Thornton AJ, Coombs KJ. The effect of quitting smoking on HDL-cholesterol - a review based on within-subject changes. *Biomarker Research.* 2013;1(1):26.

185. Moffatt RJ. Effects of cessation of smoking on serum lipids and high density lipoprotein-cholesterol. *Atherosclerosis.* 1988;74(1):85-89.

186. Rao Ch S, Subash YE. The effect of chronic tobacco smoking and chewing on the lipid profile. *J Clin Diagn Res.* 2013;7(1):31-34.

187. Gossett LK, Johnson HM, Piper ME, Fiore MC, Baker TB, Stein JH. Smoking intensity and lipoprotein abnormalities in active smokers. *J Clin Lipidol.* 2009;3(6):372-378.

188. Cullen P, Schulte H, Assmann G. Smoking, lipoproteins and coronary heart disease risk: Data from the Münster Heart Study (PROCAM). *European Heart Journal.* 1998;19(11):1632-1641.

189. Attard R, Dingli P, Doggen CJM, Cassar K, Farrugia R, Wettinger SB. The impact of passive and active smoking on inflammation, lipid profile and the risk of myocardial infarction. *Open Heart.* 2017;4(2):e000620.

190. Carreras-Torres R, Johansson M, Haycock PC, et al. Obesity, metabolic factors and risk of different histological types of lung cancer: A Mendelian randomization study. *PLOS ONE.* 2017;12(6):e0177875.

191. Jain RB. Lipid Distribution Differentials among Smokers, Non-Smokers and within Various Types of Smokers. *Annals of Clinical and Laboratory Research.* 2017;5.

192. Petruzzelli S, Puntoni R, Mimotti P, et al. Plasma 3-nitrotyrosine in cigarette smokers. *Am J Respir Crit Care Med.* 1997;156(6):1902-1907.

193. Takajo Y, Ikeda H, Haramaki N, Murohara T, Imaizumi T. Augmented oxidative stress of platelets in chronic smokers. Mechanisms of impaired platelet-derived nitric oxide bioactivity and augmented platelet aggregability. *J Am Coll Cardiol.* 2001;38(5):1320-1327.

194. Yamaguchi Y, Haginaka J, Morimoto S, Fujioka Y, Kunitomo M. Facilitated nitration and oxidation of LDL in cigarette smokers. *European Journal of Clinical Investigation.* 2005;35(3):186-193.

195. Adams T, Wan E, Wei Y, et al. Secondhand Smoking Is Associated With Vascular Inflammation. *Chest.* 2015;148(1):112-119.

196. Rytilä P, Rehn T, Ilumets H, et al. Increased oxidative stress in asymptomatic current chronic smokers and GOLD stage 0 COPD. *Respiratory Research.* 2006;7(1):69.

197. Sakano N, Takahashi N, Wang D-H, et al. Plasma 3-nitrotyrosine, urinary 8-isoprostane and 8-OHdG among healthy Japanese people. *Free Radical Research.* 2009;43(2):183-192.

198. Jin H, Webb-Robertson B-J, Peterson ES, et al. Smoking, COPD, and 3-Nitrotyrosine Levels of Plasma Proteins. *Environmental Health Perspectives.* 2011;119(9):1314-1320.

199. Korkmaz GG, Inal BB, Ortakoylu GM, et al. Changes in oxidative stress parameters and antioxidant status in lung cancer: Western blot analysis of nitrotyrosine and protein carbonyls content. *Clin Lab.* 2014;60(4):599-607.

200. Zhan X, Huang Y, Qian S. Protein Tyrosine Nitration in Lung Cancer: Current Research Status and Future Perspectives. *Current Medicinal Chemistry.* 2018;25(29):3435-3454.

201. Louhelainen N, Rytilä P, Haahtela T, Kinnula VL, Djukanović R. Persistence of oxidant and protease burden in the airways after smoking cessation. *BMC Pulmonary Medicine.* 2009;9(1):25.

202. Morita H, Ikeda H, Haramaki N, Eguchi H, Imaizumi T. Only two-week smoking cessation improves platelet aggregability and intraplatelet redox imbalance of long-term smokers. *Journal of the American College of Cardiology.* 2005;45(4):589-594.

203. Mure K, Tomono S, Mure M, et al. The Combination of Cigarette Smoking and Alcohol Consumption Synergistically Increases Reactive Carbonyl Species in Human Male Plasma. *Int J Mol Sci.* 2021;22(16).

204. Gęgotek A, Nikliński J, Žarković N, et al. Lipid mediators involved in the oxidative stress and antioxidant defence of human lung cancer cells. *Redox Biology.* 2016;9:210-219.

205. Kuiper HC, Langsdorf BL, Miranda CL, et al. Quantitation of mercapturic acid conjugates of 4-hydroxy-2-nonenal and 4-oxo-2-nonenal metabolites in a smoking cessation study. *Free Radical Biology and Medicine.* 2010;48(1):65-72.

206. Yuan JM, Carmella SG, Wang R, et al. Relationship of the oxidative damage biomarker 8-epi-prostaglandin F2α to risk of lung cancer development in the Shanghai Cohort Study. *Carcinogenesis.* 2018;39(7):948-954.

207. Dalaveris E, Kerenidi T, Katsabeki-Katsafli A, et al. VEGF, TNF-α and 8-isoprostane levels in exhaled breath condensate and serum of patients with lung cancer. *Lung Cancer.* 2009;64(2):219-225.

208. Reilly M, Delanty N, Lawson JA, FitzGerald GA. Modulation of oxidant stress in vivo in chronic cigarette smokers. *Circulation.* 1996;94(1):19-25.

209. Christensen CH, Chang JT, Rostron BL, et al. Biomarkers of Inflammation and Oxidative Stress among Adult Former Smoker, Current E-Cigarette Users-Results from Wave 1 PATH Study. *Cancer Epidemiol Biomarkers Prev.* 2021;30(10):1947-1955.

210. Oguogho A, Lupattelli G, Palumbo B, Sinzinger H. Isoprostanes quickly normalize after quitting cigarette smoking in healthy adults. *Vasa.* 2000;29(2):103-105.

211. Yao Q-H, Mei S-R, Weng Q-F, et al. Determination of urinary oxidative DNA damage marker 8-hydroxy-2′-deoxyguanosine and the association with cigarette smoking. *Talanta.* 2004;63(3):617-623.

212. Pilger A, Germadnik D, Riedel K, Meger-Kossien I, Scherer G, Rüdiger HW. Longitudinal study of urinary 8-hydroxy-2′-deoxyguanosine excretion in healthy adults. *Free Radical Research.* 2001;35(3):273-280.

213. Kulikowska-Karpińska E, Czerw K. [Estimation of 8-hydroxy-2'-deoxyguanosine (8-OHdG) concentration in the urine of cigarette smokers]. *Wiad Lek.* 2015;68(1):32-38.

214. Gackowski D, Speina E, Zielinska M, et al. Products of Oxidative DNA Damage and Repair as Possible Biomarkers of Susceptibility to Lung Cancer1. *Cancer Research.* 2003;63(16):4899-4902.

215. Calişkan-Can E, Firat H, Ardiç S, Simşek B, Torun M, Yardim-Akaydin S. Increased levels of 8-hydroxydeoxyguanosine and its relationship with lipid peroxidation and antioxidant vitamins in lung cancer. *Clin Chem Lab Med.* 2008;46(1):107-112.

216. Peddireddy V, Siva Prasad B, Gundimeda SD, Penagaluru PR, Mundluru HP. Assessment of 8-oxo-7, 8-dihydro-2′-deoxyguanosine and malondialdehyde levels as oxidative stress markers and antioxidant status in non-small cell lung cancer. *Biomarkers.* 2012;17(3):261-268.

217. Inoue M, Osaki T, Noguchi M, Hirohashi S, Yasumoto K, Kasai H. Lung Cancer Patients Have Increased 8-Hydroxydeoxyguanosine Levels in Peripheral Lung Tissue DNA. *Japanese Journal of Cancer Research.* 1998;89(7):691-695.

218. Loft S, Svoboda P, Kasai H, et al. Prospective study of 8-oxo-7,8-dihydro-2′-deoxyguanosine excretion and the risk of lung cancer. *Carcinogenesis.* 2005;27(6):1245-1250.

219. Loft S, Svoboda P, Kawai K, et al. Association between 8-oxo-7,8-dihydroguanine excretion and risk of lung cancer in a prospective study. *Free Radical Biology and Medicine.* 2012;52(1):167-172.

220. Asami S, Manabe H, Miyake J, et al. Cigarette smoking induces an increase in oxidative DNA damage, 8-hydroxydeoxyguanosine, in a central site of the human lung. *Carcinogenesis.* 1997;18(9):1763-1766.

221. Priemé H, Loft S, Klarlund M, Grønbaek K, Tønnesen P, Poulsen HE. Effect of smoking cessation on oxidative DNA modification estimated by 8-oxo-7,8-dihydro-2'-deoxyguanosine excretion. *Carcinogenesis.* 1998;19(2):347-351.

222. Inoue T, Hayashi M, Takayanagi K, Morooka S. Oxidative DNA Damage is Induced by Chronic Cigarette Smoking, but Repaired by Abstention. *Journal of Health Science.* 2003;49(3):217-220.

223. Kawasaki Y, Li Y-S, Ootsuyama Y, Nagata K, Yamato H, Kawai K. Effects of smoking cessation on biological monitoring markers in urine. *Genes and Environment.* 2020;42(1):26.

224. Agarwal P, Bagewadi A, Keluskar V, Vinuth D. Superoxide dismutase, glutathione peroxidase, and catalase antioxidant enzymes in chronic tobacco smokers and chewers: A case&#8211;control study. *Indian Journal of Dental Research.* 2019;30(2):219-225.

225. Raddam Q, Zeidan M, Asaad N, Abdulrahman M. Smoking Effects on Blood Antioxidants Level: Lactate Dehydrogenase, Catalase, Superoxide Dismutase and Glutathione Peroxidase in University Students. *Journal of Clinical & Experimental Pathology.* 2017;07.

226. Yildiz L, Kayaoğlu N, Aksoy H. The Changes of Superoxide Dismutase, Catalase and Glutathione Peroxidase Activities in Erythrocytes of Active and Passive Smokers. 2002;40(6):612-615.

227. Bolzán AD, Bianchi MS, Bianchi NO. Superoxide Dismutase, Catalase and Glutathione Peroxidase Activities in Human Blood: Influence of Sex, Age and Cigarette Smoking. *Clinical Biochemistry.* 1997;30(6):449-454.

228. Góth L. Effect of age, sex, and smoking on serum catalase activity. *Acta Biol Hung.* 1989;40(4):395-399.

229. Ahmadi-Motamayel F, Falsafi P, Goodarzi MT, Poorolajal J. Evaluation of salivary catalase, vitamin C, and alpha-amylase in smokers and non-smokers: a retrospective cohort study. *Journal of Oral Pathology & Medicine.* 2017;46(5):377-380.

230. Kondo T, Tagami S, Yoshioka A, Nishimura M, Kawakami Y. Current smoking of elderly men reduces antioxidants in alveolar macrophages. *American Journal of Respiratory and Critical Care Medicine.* 1994;149(1):178-182.

231. Ho JC-m, Zheng S, Comhair SAA, Farver C, Erzurum SC. Differential Expression of Manganese Superoxide Dismutase and Catalase in Lung Cancer1. *Cancer Research.* 2001;61(23):8578-8585.

232. Ergüder İB, Ergüder T, Özkan C, et al. Short-Term Effects of Smoking Cessation on Blood Antioxidant Parameters and Paraoxonase Activity in Healthy Asymptomatic Long-Term Cigarette Smokers. *Inhalation Toxicology.* 2006;18(8):575-579.

233. Woźniak A, Górecki D, Szpinda M, Mila-Kierzenkowska C, Woźniak B. Oxidant-Antioxidant Balance in the Blood of Patients with Chronic Obstructive Pulmonary Disease After Smoking Cessation. *Oxidative Medicine and Cellular Longevity.* 2013;2013:897075.

234. Hoidal JR, Fox RB, LeMarbe PA, Perri R, Repine JE. Altered oxidative metabolic responses in vitro of alveolar macrophages from asymptomatic cigarette smokers. *Am Rev Respir Dis.* 1981;123(1):85-89.

235. Tanni SE, Correa CR, Angeleli AY, Vale SA, Coelho LS, Godoy I. Increased production of hydrogen peroxide by peripheral blood monocytes associated with smoking exposure intensity in smokers. *Journal of Inflammation.* 2012;9(1):45.

236. Guatura SB, Martinez JA, Santos Bueno PC, Santos ML. Increased exhalation of hydrogen peroxide in healthy subjects following cigarette consumption. *Sao Paulo Med J.* 2000;118(4):93-98.

237. Lower EE, Strohofer S, Baughman RP. Bleomycin Causes Alveolar Macrophages from Cigarette Smokers to Release Hydrogen Peroxide. *The American Journal of the Medical Sciences.* 1988;295(3):193-197.

238. Baughman RP, Corser BC, Strohofer S, Hendricks D. Spontaneous hydrogen peroxide release from alveolar macrophages of some cigarette smokers. *The Journal of Laboratory and Clinical Medicine.* 1986;107(3):233-237.

239. Zieba M, Suwalski M, Kwiatkowska S, et al. Comparison of hydrogen peroxide generation and the content of lipid peroxidation products in lung cancer tissue and pulmonary parenchyma. *Respiratory Medicine.* 2000;94(8):800-805.

240. Greening AP, Lowrie DB. Extracellular release of hydrogen peroxide by human alveolar macrophages: the relationship to cigarette smoking and lower respiratory tract infections. *Clin Sci (Lond).* 1983;65(6):661-664.

241. Vilema-Enríquez G, Arroyo A, Grijalva M, Amador-Zafra RI, Camacho J. Molecular and Cellular Effects of Hydrogen Peroxide on Human Lung Cancer Cells:<i> Potential Therapeutic Implications</i>. *Oxidative Medicine and Cellular Longevity.* 2016;2016:1908164.

242. Lykkesfeldt J. Malondialdehyde as biomarker of oxidative damage to lipids caused by smoking. *Clinica Chimica Acta.* 2007;380(1):50-58.

243. Munnia A, Bonassi S, Verna A, et al. Bronchial malondialdehyde DNA adducts, tobacco smoking, and lung cancer. *Free Radical Biology and Medicine.* 2006;41(9):1499-1505.

244. Toto A, Wild P, Graille M, et al. Urinary Malondialdehyde (MDA) Concentrations in the General Population&mdash;A Systematic Literature Review and Meta-Analysis. *Toxics.* 2022;10(4):160.

245. Cobanoglu U, Demir H, Cebi A, et al. Lipid peroxidation, DNA damage and coenzyme Q10 in lung cancer patients--markers for risk assessment? *Asian Pac J Cancer Prev.* 2011;12(6):1399-1403.

246. Bartsch H, Petruzzelli S, Flora SD, et al. Carcinogen metabolism in human lung tissues and the effect of tobacco smoking: results from a case--control multicenter study on lung cancer patients. *Environmental Health Perspectives.* 1992;98:119-124.

247. Darabseh MZ, Maden-Wilkinson TM, Welbourne G, et al. Fourteen days of smoking cessation improves muscle fatigue resistance and reverses markers of systemic inflammation. *Scientific Reports.* 2021;11(1):12286.

248. Polidori MC, Mecocci P, Stahl W, Sies H. Cigarette smoking cessation increases plasma levels of several antioxidant micronutrients and improves resistance towards oxidative challenge. *British Journal of Nutrition.* 2003;90(1):147-150.

249. van Eeden SF, Hogg JC. The response of human bone marrow to chronic cigarette smoking. *Eur Respir J.* 2000;15(5):915-921.

250. Bridges RB, Fu MC, Rehm SR. Increased neutrophil myeloperoxidase activity associated with cigarette smoking. *Eur J Respir Dis.* 1985;67(2):84-93.

251. Lavi S, Prasad A, Yang EH, et al. Smoking Is Associated With Epicardial Coronary Endothelial Dysfunction and Elevated White Blood Cell Count in Patients With Chest Pain and Early Coronary Artery Disease. *Circulation.* 2007;115(20):2621-2627.

252. Vaguliene N, Zemaitis M, Lavinskiene S, Miliauskas S, Sakalauskas R. Local and systemic neutrophilic inflammation in patients with lung cancer and chronic obstructive pulmonary disease. *BMC Immunol.* 2013;14:36.

253. Van Schooten FJ, Boots AW, Knaapen AM, et al. Myeloperoxidase (MPO) −463G→A Reduces MPO Activity and DNA Adduct Levels in Bronchoalveolar Lavages of Smokers. *Cancer Epidemiology, Biomarkers & Prevention.* 2004;13(5):828-833.

254. King CC, Piper ME, Gepner AD, Fiore MC, Baker TB, Stein JH. Longitudinal Impact of Smoking and Smoking Cessation on Inflammatory Markers of Cardiovascular Disease Risk. *Arteriosclerosis, Thrombosis, and Vascular Biology.* 2017;37(2):374-379.

255. Andelid K, Bake B, Rak S, Lindén A, Rosengren A, Ekberg-Jansson A. Myeloperoxidase as a marker of increasing systemic inflammation in smokers without severe airway symptoms. *Respiratory Medicine.* 2007;101(5):888-895.

256. Padmavathi P, Vaddi DR, Maturu P, Varadacharyulu N. Smoking-Induced Alterations in Platelet Membrane Fluidity and Na+/K+-ATPase Activity in Chronic Cigarette Smokers. *Journal of atherosclerosis and thrombosis.* 2010;17:619-627.

257. Nagai K, Betsuyaku T, Kondo T, Nasuhara Y, Nishimura M. Long term smoking with age builds up excessive oxidative stress in bronchoalveolar lavage fluid. *Thorax.* 2006;61(6):496-502.

258. Pignatelli B, Li C-Q, Boffetta P, et al. Nitrated and Oxidized Plasma Proteins in Smokers and Lung Cancer Patients1. *Cancer Research.* 2001;61(2):778-784.

259. Agarwal R. Smoking, oxidative stress and inflammation: Impact on resting energy expenditure in diabetic nephropathy. *BMC Nephrology.* 2005;6(1):13.

260. Yeh C-C, Graham Barr R, Powell CA, et al. No effect of cigarette smoking dose on oxidized plasma proteins. *Environmental Research.* 2008;106(2):219-225.

261. Loffredo L, Carnevale R, Perri L, et al. NOX2-mediated arterial dysfunction in smokers: acute effect of dark chocolate. *Heart.* 2011;97(21):1776-1781.

262. Mastrangeli S, Carnevale R, Cavarretta E, et al. Predictors of oxidative stress and vascular function in an experimental study of tobacco versus electronic cigarettes: A post hoc analysis of the SUR-VAPES 1 Study. *Tob Induc Dis.* 2018;16:18.

263. Loffredo L, Zicari AM, Occasi F, et al. Passive Smoking Exacerbates Nicotinamide-Adenine Dinucleotide Phosphate Oxidase Isoform 2–Induced Oxidative Stress and Arterial Dysfunction in Children with Persistent Allergic Rhinitis. *The Journal of Pediatrics.* 2018;202:252-257.

264. Pham DM, Boussouira B, Moyal D, Nguyen QL. Oxidization of squalene, a human skin lipid: a new and reliable marker of environmental pollution studies. *International Journal of Cosmetic Science.* 2015;37(4):357-365.

265. Capitanio B, Sinagra JL, Ottaviani M, Bordignon V, Amantea A, Picardo M. Acne and smoking. *Dermato-Endocrinology.* 2009;1(3):129-135.

266. Yang Y-F, Chang Y-C, Jan Y-H, Yang C-J, Huang M-S, Hsiao M. Squalene synthase promotes the invasion of lung cancer cells via the osteopontin/ERK pathway. *Oncogenesis.* 2020;9(8):78.

267. Zhang H-Y, Li H-M, Yu Z, Yu X-y, Guo K. Expression and significance of squalene epoxidase in squamous lung cancerous tissues and pericarcinoma tissues. *Thoracic Cancer.* 2014;5(4):275-280.

268. Karademirci M, Kutlu R, Kilinc I. Relationship between smoking and total antioxidant status, total oxidant status, oxidative stress index, vit C, vit E. *The Clinical Respiratory Journal.* 2018;12(6):2006-2012.

269. Mojtaba E, Davood K, Hussein D. Lower total antioxidant capacity in smokers compare to non-smokers. Paper presented at: Biological Forum2014.

270. Jayach S, ra, Selvaraj R, Agnihotram G. Determination of serum total antioxidant capacity in male smokers and non-smokers. *National Journal of Physiology, Pharmacy and Pharmacology.* 2017;7(6):591-593.

271. Charalabopoulos K, Assimakopoulos D, Karkabounas S, Danielidis V, Kiortsis D, Evangelou A. Effects of cigarette smoking on the antioxidant defence in young healthy male volunteers. *International Journal of Clinical Practice.* 2005;59(1):25-30.

272. Bakhtiari S, Azimi S, Mehdipour M, Amini S, Elmi Z, Namazi Z. Effect of Cigarette Smoke on Salivary Total Antioxidant Capacity. *J Dent Res Dent Clin Dent Prospects.* 2015;9(4):281-284.

273. Buico A, Cassino C, Ravera M, Betta P-G, Osella D. Oxidative stress and total antioxidant capacity in human plasma. *Redox Report.* 2009;14(3):125-131.

274. Raut AM, Andure DV, Padalkar RK, Patil SM, Bhagat SS. Study of interaction of cigarette smoke with thiol (-SH) group of sulfhydryl proteins in smokers. In: IJCBR; 2019.

275. L OU, Anyadike NC, Okaforchidimma, Dioka C, Meludu SC. Evaluation of total antioxidant status, superoxide dismutase and malondialdehyde in apparently healthy active tobacco smokers in Nnewi Metropolis, South-East, Nigeria. *Journal of Scientific and Innovative Research.* 2017.

276. Gorąca A, Skibska B. Estimate of antioxidant capacity and lipid peroxidation in plasma of healthy subjects. *Central European Journal of Medicine.* 2006;1(1):23-34.

277. Ahmadkhaniha R, Yousefian F, Rastkari N. Impact of smoking on oxidant/antioxidant status and oxidative stress index levels in serum of the university students. *J Environ Health Sci Eng.* 2021;19(1):1043-1046.

278. Kurku H, Kacmaz M, Kisa U, Dogan O, Caglayan O. Acute and chronic impact of smoking on salivary and serum total antioxidant capacity. *J Pak Med Assoc.* 2015;65(2):164-169.

279. Manafa PO, Cc O, Okeke CO, et al. Assessment of Superoxide dismutase activity and total antioxidant capacity in adult male cigarette smokers in Nnewi metropolis, Nigeria. *The Journal of medical research.* 2017;3:23-26.

280. Topdag S, Aslaner A, Tataroglu C, Ilce Z. Evaluation of antioxidant capacity in lung carcinoma. *Indian Journal of Thoracic and Cardiovascular Surgery.* 2005;21(4):269-271.

281. ITO K, YANO T, MORODOMI Y, et al. Serum Antioxidant Capacity and Oxidative Injury to Pulmonary DNA in Never-smokers with Primary Lung Cancer. *Anticancer Research.* 2012;32(3):1063-1067.

282. Liu X, Zhao J, Zheng R. DNA damage of tumor-associated lymphocytes and total antioxidant capacity in cancerous patients. *Mutation Research/Genetic Toxicology and Environmental Mutagenesis.* 2003;539(1):1-8.

283. Bassey IE, Gali RM, Udoh AE. Fertility hormones and vitamin E in active and passive adult male smokers in Calabar, Nigeria. *PLOS ONE.* 2018;13(11):e0206504.

284. Marjan S, Mahmoud D, Mohammad Hassan J, Niloofar S, Abed G, Hamed M. Association of Cigarette Smoking and Serum Concentrations of Vitamins A and E in Men: A Case-Control Study. *Journal of Nutritional Sciences and Dietetics.* 2019;5(1).

285. Zhou J, Guo F, Qian Z. [Effects of cigarette smoking on antioxidant vitamin and activities of antioxidases]. *Zhonghua Yu Fang Yi Xue Za Zhi.* 1997;31(2):67-70.

286. Helmersson J, Larsson A, Vessby B, Basu S. Active smoking and a history of smoking are associated with enhanced prostaglandin F2α, interleukin-6 and F2-isoprostane formation in elderly men. *Atherosclerosis.* 2005;181(1):201-207.

287. Martens LG, Luo J, Meulmeester FL, et al. Associations between Lifestyle Factors and Vitamin E Metabolites in the General Population. *Antioxidants.* 2020;9(12):1280.

288. Huang J, Weinstein SJ, Yu K, Männistö S, Albanes D. A Prospective Study of Serum Vitamin E and 28-Year Risk of Lung Cancer. *JNCI: Journal of the National Cancer Institute.* 2019;112(2):191-199.

289. Knekt P. Vitamin E and Smoking and the Risk of Lung Cancer. *Annals of the New York Academy of Sciences.* 1993;686(1):280-287.

290. Brown AJ. Acute effects of smoking cessation on antioxidant status. *The Journal of Nutritional Biochemistry.* 1996;7(1):29-39.

291. Mittal S, Komiyama M, Ozaki Y, et al. Gingival bleeding and pocket depth among smokers and the related changes after short-term smoking cessation. *Acta Odontologica Scandinavica.* 2022;80(4):258-263.

292. Müller HP, Stadermann S. Multivariate multilevel models for repeated measures in the study of smoking effects on the association between plaque and gingival bleeding. *Clinical Oral Investigations.* 2006;10(4):311-316.

293. Krall EA, Dawson-Hughes B, Garvey AJ, Garcia RI. Smoking, Smoking Cessation, and Tooth Loss. *Journal of Dental Research.* 1997;76(10):1653-1659.

294. César Neto JB, Rosa EF, Pannuti CM, Romito GA. Smoking and periodontal tissues: a review. *Braz Oral Res.* 2012;26 Suppl 1:25-31.

295. Grossi SG, Zambon J, Machtei EE, et al. EFFECTS OF SMOKING AND SMOKING CESSATION ON HEALING AFTER MECHANICAL PERIODONTAL THERAPY. *The Journal of the American Dental Association.* 1997;128(5):599-607.

296. Sreedevi M, Ramesh A, Dwarakanath C. Periodontal Status in Smokers and Nonsmokers: A Clinical, Microbiological, and Histopathological Study. *International Journal of Dentistry.* 2012;2012:571590.

297. Erdemir EO, Duran I, Haliloglu S. Effects of smoking on clinical parameters and the gingival crevicular fluid levels of IL-6 and TNF-α in patients with chronic periodontitis. *Journal of Clinical Periodontology.* 2004;31(2):99-104.

298. Müller H-P, Stadermann S, Heinecke A. Bleeding on probing in smokers and non-smokers in a steady state plaque environment. *Clinical Oral Investigations.* 2001;5(3):177-184.

299. Shimazaki Y, Saito T, Kiyohara Y, et al. The Influence of Current and Former Smoking on Gingival Bleeding: The Hisayama Study. *Journal of Periodontology.* 2006;77(8):1430-1435.

300. Dietrich T, Bernimoulin J-P, Glynn RJ. The Effect of Cigareté Smoking on Gingival Bleeding. *Journal of Periodontology.* 2004;75(1):16-22.

301. Costa FO, Cota LOM. Cumulative smoking exposure and cessation associated with the recurrence of periodontitis in periodontal maintenance therapy: A 6-year follow-up. *Journal of Periodontology.* 2019;90(8):856-865.

302. O. Costa F, O. M. Cota L, J. P. Lages E, et al. Associations of duration of smoking cessation and cumulative smoking exposure with periodontitis. *Journal of Oral Science.* 2013;55(3):245-253.

303. Chrysanthakopoulos N. Correlation between periodontal disease indices and lung cancer in Greek adults: A case—control study. *Experimental oncology.* 2016(38,№ 1):49-53.

304. Morozumi T, Kubota T, Sato T, Okuda K, Yoshie H. Smoking cessation increases gingival blood flow and gingival crevicular fluid. *Journal of Clinical Periodontology.* 2004;31(4):267-272.

305. Nair P, Sutherland G, Palmer RM, Wilson RF, Scott DA. Gingival bleeding on probing increases after quitting smoking. *Journal of Clinical Periodontology.* 2003;30(5):435-437.

306. Preshaw PM, Heasman L, Stacey F, Steen N, McCracken GI, Heasman PA. The effect of quitting smoking on chronic periodontitis. *Journal of Clinical Periodontology.* 2005;32(8):869-879.

307. Rosa EF, Corraini P, de Carvalho VF, et al. A prospective 12-month study of the effect of smoking cessation on periodontal clinical parameters. *Journal of Clinical Periodontology.* 2011;38(6):562-571.

308. Do LG, Slade GD, Roberts-Thomson KF, Sanders AE. Smoking-attributable periodontal disease in the Australian adult population. *Journal of Clinical Periodontology.* 2008;35(5):398-404.

309. Thomson WM, Broadbent JM, Welch D, Beck JD, Poulton R. Cigarette smoking and periodontal disease among 32-year-olds: a prospective study of a representative birth cohort. *Journal of Clinical Periodontology.* 2007;34(10):828-834.

310. Ragghianti MS, Greghi SL, Lauris JR, Sant'ana AC, Passanezi E. Influence of age, sex, plaque and smoking on periodontal conditions in a population from Bauru, Brazil. *J Appl Oral Sci.* 2004;12(4):273-279.

311. Nishida N, Yamamoto Y, Tanaka M, et al. Association Between Involuntary Smoking and Salivary Markers Related to Periodontitis: A 2-Year Longitudinal Study. *Journal of Periodontology.* 2008;79(12):2233-2240.

312. Gätke D, Holtfreter B, Biffar R, Kocher T. Five-year change of periodontal diseases in the Study of Health in Pomerania (SHIP). *Journal of Clinical Periodontology.* 2012;39(4):357-367.

313. Leite FRM, Nascimento GG, Scheutz F, López R. Effect of Smoking on Periodontitis: A Systematic Review and Meta-regression. *American Journal of Preventive Medicine.* 2018;54(6):831-841.

314. Michaud DS, Lu J, Peacock-Villada AY, et al. Periodontal Disease Assessed Using Clinical Dental Measurements and Cancer Risk in the ARIC Study. *J Natl Cancer Inst.* 2018;110(8):843-854.

315. Torrungruang K, Nisapakultorn K, Sutdhibhisal S, et al. The Effect of Cigarette Smoking on the Severity of Periodontal Disease Among Older Thai Adults. *Journal of Periodontology.* 2005;76(4):566-572.

316. Rosa EF, Corraini P, Inoue G, et al. Effect of smoking cessation on non-surgical periodontal therapy: results after 24 months. *Journal of Clinical Periodontology.* 2014;41(12):1145-1153.

317. Gugnani N, Gugnani S. Can smoking cessation impact the incidence and progression of periodontitis? *Evidence-Based Dentistry.* 2020;21(4):122-123.

318. Bastiaan RJ, Waite IM. Effects of tobacco smoking on plaque development and gingivitis. *Journal of periodontology.* 1978;49(9):480-482.

319. Bergström J. Cigarette smoking as risk factor in chronic periodontal disease. *Community Dentistry and Oral Epidemiology.* 1989;17(5):245-247.

320. Feldman RS, Bravacos JS, Rose CL. Association Between Smoking Different Tobacco Products and Periodontal Disease Indexes. *Journal of Periodontology.* 1983;54(8):481-487.

321. Rösing CK, Gomes SC, Carvajal P, et al. Impact of smoking on gingival inflammation in representative samples of three South American cities. *Braz Oral Res.* 2019;33:e090.

322. Chrysanthakopoulos NA. Prevalence and associated factors of gingival recession in Greek adults. *Journal of Investigative and Clinical Dentistry.* 2013;4(3):178-185.

323. Natto S, Baljoon M, Abanmy A, Bergstrom J. Tobacco smoking and gingival health in a Saudi Arabian population. *Oral Health Prev Dent.* 2004;2(4):351-357.

324. Jenifer HD, Bhola S, Kalburgi V, Warad S, Kokatnur VM. The influence of cigarette smoking on blood and salivary super oxide dismutase enzyme levels among smokers and nonsmokers—A cross sectional study. *Journal of Traditional and Complementary Medicine.* 2015;5(2):100-105.

325. Bergström J, Preber H. The influence of cigarette smoking on the development of experimental gingivitis. *Journal of Periodontal Research.* 1986;21(6):668-676.

326. Virtanen E, Söder P-Ö, Meurman J, Andersson L, Söder B. Chronic Periodontal Disease: A Proxy of Increased Cancer Risk. *International Journal of Cancer Research.* 2013;47:2051-2784.

327. Hujoel PP, Drangsholt M, Spiekerman C, Weiss NS. An Exploration of the Periodontitis–Cancer Association. *Annals of Epidemiology.* 2003;13(5):312-316.

328. Liu KH, Hwang SJ. Effect of smoking cessation for 1 year on periodontal biomarkers in gingival crevicular fluid. *Journal of Periodontal Research.* 2016;51(3):366-375.

329. Gautam N, Shivalingesh K, Verma S, Kumar V, Saxena I. The influence of smoking on oral health and patient evaluation of tobacco cessation help from dentists working in the dental college of Bareilly city. *Journal of Addiction Medicine and Therapeutic Science.* 2022;8(1):001-005.

330. Beklen A, Yildirim BG, Mimaroglu M, Yavuz MB. The impact of smoking on oral health and patient assessment of tobacco cessation support from Turkish dentists. *Tob Induc Dis.* 2021;19:49.

331. Haffajee AD, Socransky SS. Relationship of cigarette smoking to the subgingival microbiota. *J Clin Periodontol.* 2001;28(5):377-388.

332. Grossi SG, Zambon JJ, Ho AW, et al. Assessment of Risk for Periodontal Disease. I. Risk Indicators for Attachment Loss. *Journal of Periodontology.* 1994;65(3):260-267.

333. Reibel J. Tobacco and oral diseases. Update on the evidence, with recommendations. *Med Princ Pract.* 2003;12 Suppl 1:22-32.

334. Preber H, Bergström J, Linder LE. Occurrence of periopathogens in smoker and non-smoker patients. *J Clin Periodontol.* 1992;19(9 Pt 1):667-671.

335. Stoltenberg JL, Osborn JB, Pihlstrom BL, et al. Association Between Cigarette Smoking, Bacterial Pathogens, and Periodontal Status. *Journal of Periodontology.* 1993;64(12):1225-1230.

336. Boström L, Bergström J, Dahlén G, Linder LE. Smoking and subgingival microflora in periodontal disease. *J Clin Periodontol.* 2001;28(3):212-219.

337. Shi J, Yang Y, Xie H, et al. Association of oral microbiota with lung cancer risk in a low-income population in the Southeastern USA. *Cancer Causes & Control.* 2021;32(12):1423-1432.

338. Mai X, Genco RJ, LaMonte MJ, et al. Periodontal Pathogens and Risk of Incident Cancer in Postmenopausal Females: The Buffalo OsteoPerio Study. *Journal of Periodontology.* 2016;87(3):257-267.

339. Yang J, Mu X, Wang Y, et al. Dysbiosis of the Salivary Microbiome Is Associated With Non-smoking Female Lung Cancer and Correlated With Immunocytochemistry Markers. *Frontiers in Oncology.* 2018;8.

340. Hosgood HD, Cai Q, Hua X, et al. Variation in oral microbiome is associated with future risk of lung cancer among never-smokers. *Thorax.* 2021;76(3):256-263.

341. Zhou B, Lu J, Beck JD, et al. Periodontal and other oral bacteria and risk of lung cancer in the Atherosclerosis Risk in Communities (ARIC) Study. *Cancer Epidemiology, Biomarkers & Prevention.* 2022.

342. Cai Q, Long J, Xie H, et al. Abstract 3455: Association of oral microbiome with lung cancer risk: Results from the Southern Community Cohort Study. *Cancer Research.* 2016;76(14_Supplement):3455-3455.

343. Yan X, Yang M, Liu J, et al. Discovery and validation of potential bacterial biomarkers for lung cancer. *Am J Cancer Res.* 2015;5(10):3111-3122.

344. Fullmer SC, Preshaw PM, Heasman PA, Kumar PS. Smoking cessation alters subgingival microbial recolonization. *J Dent Res.* 2009;88(6):524-528.

345. Moimaz S, Zina L, Saliba O, Garbin C. Smoking and periodontal disease: Clinical evidence for an association. *Primary Dental Care.* 2010;17.

346. Rudziński R, Banach J. [The effect of tobacco smoke on the course and severity of inflammation in periodontal tissues]. *Ann Acad Med Stetin.* 2011;57(1):88-95.

347. Zini A, Sgan-Cohen HD, Marcenes W. Socio-economic position, smoking, and plaque: a pathway to severe chronic periodontitis. *Journal of Clinical Periodontology.* 2011;38(3):229-235.

348. Swenson HM. The effect of cigarette smoking on plaque formation. *Journal of periodontology.* 1979;50(3):146-147.

349. Macgregor IDM, Edgar WM, Greenwood AR. Effects of cigarette smoking on the rate of plaque formation. *Journal of Clinical Periodontology.* 1985;12(1):35-41.

350. Chrysanthakopoulos NA. Periodontal disease-cancer association and the specific role of periodontal disease in lung cancer pathogenesis. *Mathews Journal of Dentistry.* 2018;3(1):1-11.

351. Rad M, Kakoie S, Niliye Brojeni F, Pourdamghan N. Effect of Long-term Smoking on Whole-mouth Salivary Flow Rate and Oral Health. *J Dent Res Dent Clin Dent Prospects.* 2010;4(4):110-114.

352. Kerdvongbundit V, Wikesjö UME. Effect of Smoking on Periodontal Health in Molar Teeth. *Journal of Periodontology.* 2000;71(3):433-437.

353. Bergström J, Ellasson S. Noxious effect of cigarette smoking on periodontal health. *Journal of Periodontal Research.* 1987;22(6):513-517.

354. Bretz W. Smokers with adequate oral hygiene habits who visit the dentist at least twice a year have a greater prevalence and severity of periodontal disease at mandibular molar teeth than never-smokers with similar oral health practices. *Journal of Evidence Based Dental Practice.* 2003;3(2):98-99.

355. Muszyński P, Polańska K, Hanke W. [Effects of smoking on periodontal tissues and benefits of tobacco quitting]. *Przegl Lek.* 2014;71(11):648-653.

356. Kaleem MM, Nazir R, Manzoor MA. ASSOCIATION BETWEEN CIGARATTE SMOKING AND PERIODONTITIS IN PAKISTANI POPULATION. 2009.

357. Gloria C, Ramón J-M, Echeverría J-J. Effects of smoking on periodontal tissues. *Journal of Clinical Periodontology.* 2002;29(8):771-776.

358. Krall EA, Garvey AJ, Garcia RI. ALVEOLAR BONE LOSS AND TOOTH LOSS IN MALE CIGAR AND PIPE SMOKERS. *The Journal of the American Dental Association.* 1999;130(1):57-64.

359. Arora M, Weuve J, Fall K, Pedersen NL, Mucci LA. An Exploration of Shared Genetic Risk Factors Between Periodontal Disease and Cancers: A Prospective Co-Twin Study. *American Journal of Epidemiology.* 2010;171(2):253-259.

360. Jawzali JI. Association between salivary sialic acid and periodontal health status among smokers. *Saudi Dent J.* 2016;28(3):124-135.

361. Silver KJ, Sachs DP, Hottel TL. Gingival response to nicotine polacrilex. *The Journal of the American Dental Association.* 1989;118(1):53-56.

362. van der Plas A, Pouly S, de La Bourdonnaye G, Ng WT, Baker G, Lüdicke F. Influence of smoking and smoking cessation on levels of urinary 11-dehydro thromboxane B2. *Toxicology Reports.* 2018;5:561-567.

363. Cathcart M-C, Gately K, Cummins R, Kay E, O'Byrne KJ, Pidgeon GP. Examination of thromboxane synthase as a prognostic factor and therapeutic target in non-small cell lung cancer. *Molecular Cancer.* 2011;10(1):25.

364. Chen GG, Lee TW, Yip JHY, et al. Increased thromboxane B2 levels are associated with lipid peroxidation and Bcl-2 expression in human lung carcinoma. *Cancer Letters.* 2006;234(2):193-198.

365. Grafetstätter M, Hüsing A, González Maldonado S, et al. Plasma Fibrinogen and sP-Selectin are Associated with the Risk of Lung Cancer in a Prospective Study. *Cancer Epidemiology, Biomarkers & Prevention.* 2019;28(7):1221-1227.

366. dos Santos Silva I, De Stavola BL, Pizzi C, Meade TW. Circulating levels of coagulation and inflammation markers and cancer risks: individual participant analysis of data from three long-term cohorts. *International Journal of Epidemiology.* 2010;39(3):699-709.

367. Allin KH, Bojesen SE, Nordestgaard BG. Inflammatory biomarkers and risk of cancer in 84,000 individuals from the general population. *International Journal of Cancer.* 2016;139(7):1493-1500.

368. Hammett CJK, Prapavessis H, Baldi JC, et al. Variation in Blood Levels of Inflammatory Markers Related and Unrelated to Smoking Cessation in Women. *Preventive Cardiology.* 2007;10(2):68-75.

369. Simpson AJ, Gray RS, Moore NR, Booth NA. THE EFFECTS OF CHRONIC SMOKING ON THE FIBRINOLYTIC POTENTIAL OF PLASMA AND PLATELETS. *British Journal of Haematology.* 1997;97(1):208-213.

370. Scarabin P-Y, Aillaud M-F, Amouyel P, et al. Associations of Fibrinogen, Factor VII and PAI-1 with Baseline Findings among 10,500 Male Participants in a Prospective Study of Myocardial Infarction. *Thromb Haemost.* 1998;80(11):749-756.

371. Sasaki A, Kurisu A, Ohno M, Ikeda Y. Overweight/Obesity, Smoking, and Heavy Alcohol Consumption Are Important Determinants of Plasma PAI-1 Levels in Healthy Men. *The American Journal of the Medical Sciences.* 2001;322(1):19-23.

372. Ozaki K, Hori T, Ishibashi T, Nishio M, Aizawa Y. Effects of chronic cigarette smoking on endothelial function in young men. *Journal of Cardiology.* 2010;56(3):307-313.

373. Liu EE, Suthahar N, Paniagua SM, et al. Association of Cardiometabolic Disease With Cancer in the Community. *JACC: CardioOncology.* 2022;4(1):69-81.

374. Sotiropoulos GP, Kotopouli M, Karampela I, et al. Circulating plasminogen activator inhibitor-1 activity: a biomarker for resectable non-small cell lung cancer? *J buon.* 2019;24(3):943-954.

375. Salden M, Splinter TAW, Peters HA, et al. The urokinase-type plasminogen activator system in resected non-small-cell lung cancer. *Annals of Oncology.* 2000;11(3):327-332.

376. Minami J, Todoroki M, Yoshii M, et al. Effects of smoking cessation or alcohol restriction on metabolic and fibrinolytic variables in Japanese men. *Clin Sci (Lond).* 2002;103:117-122.

377. Varol E, Icli A, Kocyigit S, Erdogan D, Ozaydin M, Dogan A. Effect of Smoking Cessation on Mean Platelet Volume. *Clinical and Applied Thrombosis/Hemostasis.* 2012;19(3):315-319.

378. Gitte RN. Effect of Cigarette Smoking on Plasma Fibrinogen and Platelet Count. *Asian Journal of Medical Sciences.* 2012;2(3):181-184.

379. Erikssen J, Hellem A, Stormorken H. Chronic Effect of Smoking on Platelet Count and “Platelet Adhesiveness” in Presumably Healthy Middle-Aged Men. *Thromb Haemost.* 1977;38(07):0606-0611.

380. Tell GS, Grimm RH, Vellar OD, Theodorsen L. The relationship of white cell count, platelet count, and hematocrit to cigarette smoking in adolescents: the Oslo Youth Study. *Circulation.* 1985;72(5):971-974.

381. Aghaji M, Nnabuko R, Uzuegbunam C, Oyeka IC. The relationship of white blood cell and platelet counts to cigarette smoking in adult Nigerians. *Cent Afr J Med.* 1990;36(11):273-278.

382. Ghahremanfard F, Semnani V, Ghorbani R, Malek F, Behzadfar A, Zahmatkesh M. Effects of cigarette smoking on morphological features of platelets in healthy men. *Saudi Med J.* 2015;36(7):847-850.

383. Pujani M, Chauhan V, Singh K, Rastogi S, Agarwal C, Gera K. The effect and correlation of smoking with platelet indices, neutrophil lymphocyte ratio and platelet lymphocyte ratio. *Hematology, transfusion and cell therapy.* 2020;43.

384. Zhu Y, Wei Y, Zhang R, et al. Elevated Platelet Count Appears to Be Causally Associated with Increased Risk of Lung Cancer: A Mendelian Randomization Analysis. *Cancer Epidemiology, Biomarkers & Prevention.* 2019;28(5):935-942.

385. Mounce LT, Hamilton W, Bailey SE. Cancer incidence following a high-normal platelet count: cohort study using electronic healthcare records from English primary care. *British Journal of General Practice.* 2020;70(698):e622-e628.

386. Zhang X, Ran Y. Prognostic role of elevated platelet count in patients with lung cancer: a systematic review and meta-analysis. *Int J Clin Exp Med.* 2015;8(4):5379-5387.

387. Gonzalez Barcala FJ, Garcia Prim JM, Moldes Rodriguez M, et al. Platelet count: association with prognosis in lung cancer. *Medical Oncology.* 2010;27(2):357-362.

388. Green MS, Peled I, Najenson T. Gender differences in platelet count and its association with cigarette smoking in a large cohort in Israel. *Journal of Clinical Epidemiology.* 1992;45(1):77-84.

389. Yarlioglues M, Ardic I, Dogdu O, et al. The acute effects of passive smoking on mean platelet volume in healthy volunteers. *Angiology.* 2012;63(5):353-357.

390. Bain BJ, Rothwell M, Feher MD, Robinson R, Brown J, Sever PS. Acute changes in haematological parameters on cessation of smoking. *J R Soc Med.* 1992;85(2):80-82.

391. Neubauer H, Setiadi P, Pinto A, et al. Upregulation of platelet CD40, CD40 ligand (CD40L) and P-Selectin expression in cigarette smokers: a flow cytometry study. *Blood Coagulation & Fibrinolysis.* 2009;20(8).

392. Harding SA, Sarma J, Josephs DH, et al. Upregulation of the CD40/CD40 Ligand Dyad and Platelet-Monocyte Aggregation in Cigarette Smokers. *Circulation.* 2004;109(16):1926-1929.

393. Kayrak M, Bacaksiz A, Ulgen MS, et al. Plasma concentrations of soluble CD40 ligand in smokers with acute myocardial infarction: a pilot study. *Heart and Vessels.* 2011;26(2):131-137.

394. Roselli M, Mineo TC, Basili S, et al. Soluble CD40 ligand plasma levels in lung cancer. *Clin Cancer Res.* 2004;10(2):610-614.

395. Mu C-Y, Qin P-X, Qu Q-X, Chen C, Huang J-A. Soluble CD40 in plasma and malignant pleural effusion with non-small cell lung cancer: A potential marker of prognosis. *Chronic Diseases and Translational Medicine.* 2015;1(1):36-41.

396. Sabel MS, Yamada M, Kawaguchi Y, Chen F-A, Takita H, Bankert RB. CD40 expression on human lung cancer correlates with metastatic spread. *Cancer Immunology, Immunotherapy.* 2000;49(2):101-108.

397. Adatia A, Wahab M, Shahid I, Moinuddin A, Killian KJ, Satia I. Effects of cigarette smoke exposure on pulmonary physiology, muscle strength and exercise capacity in a retrospective cohort with 30,000 subjects. *PLOS ONE.* 2021;16(6):e0250957.

398. Schwartz DA, Merchant RK, Helmers RA, Gilbert SR, Dayton CS, Hunninghake GW. The influence of cigarette smoking on lung function in patients with idiopathic pulmonary fibrosis. *Am Rev Respir Dis.* 1991;144(3 Pt 1):504-506.

399. Sill J. The Effects of Smoking on Pulmonary Function Testing. *CHEST.* 2016;149(4):A591.

400. van Ganse WF, Ferris BG, Cotes JE. Cigarette Smoking and Pulmonary Diffusing Capacity (Transfer Factor). *American Review of Respiratory Disease.* 1972;105(1):30-41.

401. Frans A, Stănescu DC, Veriter C, Clerbaux T, Brasseur L. Smoking and pulmonary diffusing capacity. *Scand J Respir Dis.* 1975;56(3):165-183.

402. Mahajan KK, Mahajan SK, Mishra N. Effect of Exercise on Lung Transfer Factor for CO in Smokers. *Respiration.* 1991;58(3-4):167-170.

403. Knudson RJ, Kaltenborn WT, Burrows B. The Effects of Cigarette Smoking and Smoking Cessation on the Carbon Monoxide Diffusing Capacity of the Lung in Asymptomatic Subjects. *American Review of Respiratory Disease.* 1989;140(3):645-651.

404. de Torres JP, Marín JM, Casanova C, et al. Lung Cancer in Patients with Chronic Obstructive Pulmonary Disease. *American Journal of Respiratory and Critical Care Medicine.* 2011;184(8):913-919.

405. Yoo H, Jeong BH, Chung MJ, Lee KS, Kwon OJ, Chung MP. Risk factors and clinical characteristics of lung cancer in idiopathic pulmonary fibrosis: a retrospective cohort study. *BMC Pulm Med.* 2019;19(1):149.

406. Sansores RH, Pare P, Abboud RT. Effect of Smoking Cessation on Pulmonary Carbon Monoxide Diffusing Capacity and Capillary Blood Volume. *American Review of Respiratory Disease.* 1992;146(4):959-964.

407. Shaker SB, Stavngaard T, Laursen LC, Stoel BC, Dirksen A. Rapid Fall in Lung Density Following Smoking Cessation in COPD. *COPD: Journal of Chronic Obstructive Pulmonary Disease.* 2011;8(1):2-7.

408. Bhargava EK, Khaliq F. Effect of paternal smoking on the pulmonary functions of adolescent males. *Indian J Physiol Pharmacol.* 2008;52(4):413-419.

409. Rexhepi A, Brestovci B. Influence Of Smoking And Physical Activity On Pulmonary Function. *Internet Journal of Pulmonary Medicine.* 2008;11.

410. Kougias M, Vardavas CI, Anagnostopoulos N, et al. The acute effect of cigarette smoking on the respiratory function and FENO production among young smokers. *Experimental Lung Research.* 2013;39(8):359-364.

411. Michels A, Decoster K, Derde L, Vleurinck C, Van de Woestijne KP. Influence of posture on lung volumes and impedance of respiratory system in healthy smokers and nonsmokers. *Journal of Applied Physiology.* 1991;71(1):294-299.

412. Sobol BJ, Van Voorhies L, Emirgil C. Detection of acute effects of cigarette smoking on airway dynamics: A critical and comparative study of pulmonary function tests. *Thorax.* 1977;32(3):312-316.

413. Zamarrón E, Prats E, Tejero E, et al. Static lung hyperinflation is an independent risk factor for lung cancer in patients with chronic obstructive pulmonary disease. *Lung Cancer.* 2019;128:40-46.

414. Crooks CJ, West J, Morling JR, et al. Pulse oximeter measurement error of oxygen saturation in patients with SARS-CoV-2 infection stratified by smoking status. *European Respiratory Journal.* 2022:2201190.

415. Dawley HH, Ellithorpe DB, Tretola R. Aversive smoking: Carboxyhemoglobin levels before and after rapid smoking. *Journal of Behavior Therapy and Experimental Psychiatry.* 1976;7(1):13-15.

416. Vold ML, Aasebø U, Melbye H. Low FEV1, smoking history, and obesity are factors associated with oxygen saturation decrease in an adult population cohort. *Int J Chron Obstruct Pulmon Dis.* 2014;9:1225-1233.

417. Tirlapur VG, Gicheru K, Charalambous BM, Evans PJ, Mir MA. Packed cell volume, haemoglobin, and oxygen saturation changes in healthy smokers and non-smokers. *Thorax.* 1983;38(10):785-787.

418. Zeng X, Ren Y, Wu K, et al. Association between smoking behavior and obstructive sleep apnea: A systematic review and meta-analysis. *Nicotine & Tobacco Research.* 2022:ntac126.

419. Kendzerska T, Povitz M, Leung RS, et al. Obstructive Sleep Apnea and Incident Cancer: A Large Retrospective Multicenter Clinical Cohort Study. *Cancer Epidemiology, Biomarkers & Prevention.* 2021;30(2):295-304.

420. Kendzerska T, Leung RS, Hawker G, Tomlinson G, Gershon AS. Obstructive sleep apnea and the prevalence and incidence of cancer. *Canadian Medical Association Journal.* 2014;186(13):985-992.

421. Campos-Rodriguez F, Martinez-Garcia MA, Martinez M, et al. Association between obstructive sleep apnea and cancer incidence in a large multicenter Spanish cohort. *Am J Respir Crit Care Med.* 2013;187(1):99-105.

422. Bugter O, van Brummelen SE, van der Leest KH, et al. Towards the Optical Detection of Field Cancerization in the Buccal Mucosa of Patients with Lung Cancer. *Translational Oncology.* 2019;12(12):1533-1538.

423. Pezzuto A, Carico E. Effectiveness of smoking cessation in smokers with COPD and nocturnal oxygen desaturation: Functional analysis. *The Clinical Respiratory Journal.* 2020;14(1):29-34.

424. Crowley TJ, Macdonald MJ, Walter MI. Behavioral anti-smoking trial in chronic obstructive pulmonary disease patients. *Psychopharmacology.* 1995;119(2):193-204.

425. Watson A, Joyce H, Pride NB. Changes in carbon monoxide transfer over 22 years in middle-aged men. *Respiratory Medicine.* 2000;94(11):1103-1108.

426. Clark K, Wardrobe-Wong N, Elliott J, Gill P, Tait N, Snashall P. Cigarette smoke inhalation and lung damage in smoking volunteers. *European Respiratory Journal.* 1998;12(2):395-399.

427. Watson A, Joyce H, Hopper L, Pride NB. Influence of smoking habits on change in carbon monoxide transfer factor over 10 years in middle aged men. *Thorax.* 1993;48(2):119-124.

428. Dhariwal J, Tennant RC, Hansell DM, et al. Smoking cessation in COPD causes a transient improvement in spirometry and decreases micronodules on high-resolution CT imaging. *Chest.* 2014;145(5):1006-1015.

429. Lapperre TS, Postma DS, Gosman MME, et al. Relation between duration of smoking cessation and bronchial inflammation in COPD. *Thorax.* 2006;61(2):115-121.

430. Hensler NM, Giron DJ. Pulmonary Physiological Measurements in Smokers and Nonsmokers. *JAMA.* 1963;186(10):885-889.

431. York EL, Jones RL. Effects of Smoking on Regional Residual Volume in Young Adults. *Chest.* 1981;79(1):12-15.

432. Paoletti P, Viegi G, Carrozzi L, et al. Residual Volume in a General Population: Effects of Body Size, Age, Cigarette Smoking, and Respiratory Symptoms. *Chest.* 1992;102(4):1209-1215.

433. Bai JW, Chen XX, Liu S, Yu L, Xu JF. Smoking cessation affects the natural history of COPD. *Int J Chron Obstruct Pulmon Dis.* 2017;12:3323-3328.

434. Nemery B, Moavero NE, Brasseur L, Stănescu DC, Pahulycz C, Veriter C. Changes In Lung Function After Smoking Cessation: An Assessment from a Cross-Sectional Survey. *American Review of Respiratory Disease.* 1982;125(1):122-124.

435. Amaral JLM, Lopes AJ, Jansen JM, Faria ACD, Melo PL. An improved method of early diagnosis of smoking-induced respiratory changes using machine learning algorithms. *Computer Methods and Programs in Biomedicine.* 2013;112(3):441-454.

436. Faria ACD, da Costa AA, Lopes AJ, Jansen JM, de Melo PL. Forced oscillation technique in the detection of smoking-induced respiratory alterations: diagnostic accuracy and comparison with spirometry. *Clinics.* 2010;65(12):1295-1304.

437. Faria ACD, Lopes AJ, Jansen JM, Melo PL. Evaluating the forced oscillation technique in the detection of early smoking-induced respiratory changes. *BioMedical Engineering OnLine.* 2009;8(1):22.

438. Shinke H, Yamamoto M, Hazeki N, Kotani Y, Kobayashi K, Nishimura Y. Visualized changes in respiratory resistance and reactance along a time axis in smokers: A cross-sectional study. *Respiratory Investigation.* 2013;51(3):166-174.

439. Bhattarai P, Myers S, Chia C, et al. Clinical Application of Forced Oscillation Technique (FOT) in Early Detection of Airway Changes in Smokers. *Journal of Clinical Medicine.* 2020;9(9):2778.

440. Oostveen E, Boda K, van der Grinten CPM, et al. Respiratory impedance in healthy subjects: baseline values and bronchodilator response. *European Respiratory Journal.* 2013;42(6):1513-1523.

441. Freund KM, Belanger AJ, D'Agostino RB, Kannel WB. The health risks of smoking the framingham study: 34 years of follow-up. *Annals of Epidemiology.* 1993;3(4):417-424.

442. Yang SC. [Relationship between smoking habits and lung function changes with conventional spirometry]. *J Formos Med Assoc.* 1993;92 Suppl 4:S225-231.

443. Roca J, Burgos F, Sunyer J, et al. References values for forced spirometry. Group of the European Community Respiratory Health Survey. *European Respiratory Journal.* 1998;11(6):1354-1362.

444. Lee PN, Fry JS. Systematic review of the evidence relating FEV1decline to giving up smoking. *BMC Medicine.* 2010;8(1):84.

445. Oelsner EC, Balte PP, Bhatt SP, et al. Lung function decline in former smokers and low-intensity current smokers: a secondary data analysis of the NHLBI Pooled Cohorts Study. *The Lancet Respiratory Medicine.* 2020;8(1):34-44.

446. Anthonisen NR, Connett JE, Murray RP. Smoking and Lung Function of Lung Health Study Participants after 11 Years. *American Journal of Respiratory and Critical Care Medicine.* 2002;166(5):675-679.

447. Bajentri AL, Veeranna N, Dixit PD, Kulkarni SB. Effect of 2-5 years of tobacco smoking on ventilatory function tests. *J Indian Med Assoc.* 2003;101(2):96-97, 108.

448. Boezen H, Schouten J, Postma D, Rijcken B. Distribution of peak expiratory flow variability by age, gender and smoking habits in a random population sample aged 20-70 yrs. *European Respiratory Journal.* 1994;7(10):1814-1820.

449. Seppänen A. Comparison of different kinds of tests in the evaluation of lung function among healthy smokers and nonsmokers. *Ann Clin Res.* 1977;9(5):275-280.

450. Yoon YJ, Lee MS, Jang KW, Ahn JB, Hurh K, Park E-C. Association between smoking cessation and obstructive spirometry pattern among Korean adults aged 40–79 years. *Scientific Reports.* 2021;11(1):18667.

451. Mannino DM, Aguayo SM, Petty TL, Redd SC. Low Lung Function and Incident Lung Cancer in the United States: Data From the First National Health and Nutrition Examination Survey Follow-up. *Archives of Internal Medicine.* 2003;163(12):1475-1480.

452. Su Z, Jiang Y, Li C, et al. Relationship between lung function and lung cancer risk: a pooled analysis of cohorts plus Mendelian randomization study. *Journal of Cancer Research and Clinical Oncology.* 2021;147(10):2837-2849.

453. Wasswa-Kintu S, Gan WQ, Man SFP, Pare PD, Sin DD. Relationship between reduced forced expiratory volume in one second and the risk of lung cancer: a systematic review and meta-analysis. *Thorax.* 2005;60(7):570-575.

454. Nomura A, Stemmermann GN, Chyou P-H, Marcus EB, Buist AS. Prospective Study of Pulmonary Function and Lung Cancer. *American Review of Respiratory Disease.* 1991;144(2):307-311.

455. Fry JS, Hamling JS, Lee PN. Systematic review with meta-analysis of the epidemiological evidence relating FEV1decline to lung cancer risk. *BMC Cancer.* 2012;12(1):498.

456. Cook NR, Evans DA, Scherr PA, Speizer FE, Taylor JO, Hennekens CH. Peak Expiratory Flow Rate and 5-Year Mortality in an Elderly Population. *American Journal of Epidemiology.* 1991;133(8):784-794.

457. Ge H, Jiang Z, Huang Q, Zhu M, Yang J. Correlation between Pulmonary Function Indexes and Survival Time in Patients with Advanced Lung Cancer. *Zhongguo Fei Ai Za Zhi.* 2013;16(7):359-363.

458. Smith M, Zhou M, Wang L, Peto R, Yang G, Chen Z. Peak flow as a predictor of cause-specific mortality in China: results from a 15-year prospective study of ∼170 000 men. *International Journal of Epidemiology.* 2013;42(3):803-815.

459. Gold DR, Wang X, Wypij D, Speizer FE, Ware JH, Dockery DW. Effects of Cigarette Smoking on Lung Function in Adolescent Boys and Girls. *New England Journal of Medicine.* 1996;335(13):931-937.

460. Medabala T, B NR, Mohesh MIG, Kumar MP. Effect of cigarette and cigar smoking on peak expiratory flow rate. *J Clin Diagn Res.* 2013;7(9):1886-1889.

461. Pride NB. Smoking cessation: effects on symptoms, spirometry and future trends in COPD. *Thorax.* 2001;56(suppl 2):ii7-ii10.

462. Buist AS, Nagy JM, Sexton GJ. The Effect of Smoking Cessation on Pulmonary Function: A 30-Month Follow-up of Two Smoking Cessation Clinics. *American Review of Respiratory Disease.* 1979;120(4):953-957.

463. Corbin RP, Loveland M, Martin RR, Macklem PT. A Four-Year Follow-up Study of Lung Mechanics in Smokers. *American Review of Respiratory Disease.* 1979;120(2):293-304.

464. Blackburn H, Brozek J, Taylor H. LUNG VOLUME IN SMOKERS AND NONSMOKERS. *Annals of Internal Medicine.* 1959;51(1):68-77.

465. Suzuki S, Sasaki H, Takishima T. Effects of Smoking on Dynamic Compliance and Respiratory Resistance. *Archives of Environmental Health: An International Journal.* 1983;38(3):133-137.

466. Yang SC, Yang SP. Bronchial responsiveness and lung function related to cigarette smoking and smoking cessation. *Chang Gung Med J.* 2002;25(10):645-655.

467. Làndsér FJ, Clément J, Woestijne KPVd. Normal Values of Total Respiratory Resistance and Reactance Determined by Forced Oscillations: Influence of Smoking. *CHEST.* 1982;81(5):586-591.

468. Gimeno F, van der Weele LT, Koëter GH, van Altena R. Forced oscillation technique. Reference values for total respiratory resistance obtained with the Siemens Siregnost FD5. *Ann Allergy.* 1992;68(2):155-158.

469. Peslin R, Hannhart B, Pino J. [Mechanical impedance of the chest in smokers and non-smokers (author's transl)]. *Bull Eur Physiopathol Respir.* 1981;17(1):93-105.
